# Consumer Attitudes, Knowledge, and Consumption Behaviours Toward Game Meat in Romania: Evidence from an Online Convenience Sample

**DOI:** 10.3390/foods15183179

**Published:** 2026-09-08

**Authors:** Răzvan-Tudor Pătrînjan, Adriana Morar, Viorel Herman, Emil Tîrziu, Alexandra Ban-Cucerzan, Sebastian-Alexandru Popa, Bianca-Luiza Ghițan, Daiana-Ionela Cocoș, Kálmán Imre

**Affiliations:** 1 Department of Animal Production and Veterinary Public Health, Faculty of Veterinary Medicine, University of Life Sciences “King Mihai I” from Timișoara, Calea Aradului 119, 300645 Timișoara, Romania; adrianamorar@usvt.ro (A.M.); emiltarziu@yahoo.com (E.T.); alexandra.ban-cucerzan@usvt.ro (A.B.-C.); sebastian.popa@usvt.ro (S.-A.P.); bianca.ghitan.iosud@usvt.ro (B.-L.G.); 2 Department of Infectious Disease and Preventive Medicine, Faculty of Veterinary Medicine, University of Life Sciences “King Mihai I” from Timișoara, Calea Aradului 119, 300645 Timișoara, Romania; viorel.herman@fmvt.ro; 3 Department of Pharmacy and Pharmacology, Faculty of Veterinary Medicine, University of Life Sciences “King Mihai I” from Timișoara, Calea Aradului 119, 300645 Timișoara, Romania; daiana.cocos.fmv@usvt.ro

**Keywords:** game meat consumption, consumer behaviour, food choice, wildlife products, food perception, Romania

## Abstract

Game meat is increasingly discussed as a nutrient-rich alternative source of animal protein, although its consumption remains constrained by limited availability, consumer familiarity, and practical or perceptual barriers. This study assessed attitudes, knowledge, perceptions, and consumption behaviours related to game meat among respondents recruited through an online convenience survey in Romania. Following exclusion of three respondents who did not meet the predefined age criterion, 640 participants were included in the analysis. Data were analysed using descriptive and inferential statistics, multiple correspondence analysis (MCA) with exploratory clustering, and multivariable binary logistic regression. Overall, 75.8% of respondents (95% CI [72.3–78.9]) reported having previously consumed game meat, although consumption was predominantly infrequent. Having family members or friends involved in hunting showed the strongest adjusted association with previous consumption (OR = 4.81, 95% CI [2.59–8.94]), while older age and higher self-rated information were also associated with higher adjusted odds of previous consumption. Exploratory MCA-based clustering suggested three broad respondent profiles characterised by differing levels of familiarity, previous consumption, purchasing knowledge, and perceptions of game meat. Among non-consumers, 50.0% reported willingness to try game meat in the future, although the corresponding multivariable model had limited explanatory power. The principal reported barriers among non-consumers were lack of opportunity (63.2%), discomfort with the idea of eating wild animals (43.4%), and ethical concerns regarding hunting (34.2%). These findings highlight potential considerations for producers, retailers, and policy makers, including market accessibility, consumer information, culinary guidance, and transparent communication regarding product origin and safety. Given the young, student-dominated convenience sample, the findings primarily reflect the perspectives of younger, highly educated respondents in Romania.

## 1. Introduction

Game meat, derived from free-ranging wild animals such as wild boar, roe deer, red deer, and game birds, occupies a unique position within contemporary food systems. Unlike meat produced through intensive livestock farming, game meat originates from wildlife management and regulated hunting practices and has attracted increasing scientific and consumer interest as a potentially sustainable source of high-quality animal protein. Owing to its naturally lean composition, favourable fatty acid profile, high nutritional value, and limited reliance on external feed resources, antibiotics, and housing infrastructure, game meat is increasingly regarded as a food that combines nutritional benefits with comparatively low production inputs [1,2,3].

The growing interest in alternative and sustainably produced animal proteins has further increased attention toward game meat, particularly among consumers seeking foods perceived as natural, minimally processed, and environmentally responsible.

Against a background of intensifying debate over the environmental footprint of conventional meat and growing consumer interest in “natural” and locally sourced foods, game meat has attracted renewed attention as a niche protein with potential nutritional and sustainability advantages [4,5,6,7,8].

Ultimately, the successful integration of game meat into modern food systems depends not only on its nutritional and environmental characteristics but also on consumer acceptance, purchasing behaviour, and confidence in its safety and quality. Consequently, understanding the factors that influence consumer choices has become an important component of sustainable food system research. These marked differences in availability and consumption patterns have stimulated a growing body of consumer research aimed at understanding the drivers and barriers influencing acceptance of game meat across different European populations.

Despite these attributes, game meat remains a marginal component of the European diet. Its supply is seasonal and quantitatively limited, its distribution through mainstream retail is uneven, and consumer familiarity varies widely across countries and social groups [2,9]. Studies across the continent consistently describe a small core of regular consumers, often linked to hunting households, surrounded by a larger group of occasional or non-consumers whose behaviour is shaped by limited access, uncertainty about preparation, price perceptions, and concerns about food safety and hunting ethics [5,9,10,11,12]. Perceptions are heterogeneous: the same product is regarded by some consumers as a healthy, natural delicacy that fulfils nutritional expectations [6] and by others as difficult to source, complicated to cook, or potentially unsafe or unacceptable due to strong wild flavour characteristics [1]. Furthermore, consumer preferences are strongly driven by prior product knowledge and explicit communication regarding whether wild meat is hunted or farmed [13].

Consumer research on game meat has expanded over the past decade but remains geographically concentrated. Detailed studies exist for Poland [9], Italy [5], Spain [14], the Czech Republic [7], Croatia [11,15], and Portugal [12], and a recent literature review has synthesised the evidence base [2]. Romania, however, is under-represented. Although Romanian research has documented the nutritional composition and sensory quality of game meat [3,16], studies from Romania and across Europe have also identified foodborne pathogens and antimicrobial-resistance concerns in wild ungulates [10,17], while the consumer side remains under-examined. How respondents in Romania perceive game meat, how often and where they obtain it, what deters them, and which sociodemographic groups are most and least engaged have not been documented in a structured survey. This gap is relevant not only locally but also for the wider question of how game meat markets develop in Central and Eastern European countries with a hunting heritage but limited formal retail infrastructure.

The present study addresses this gap through a cross-sectional survey of respondents in Romania. Its objectives were: (i) to describe consumer awareness, consumption frequency, purchasing habits and channels, and the motivations and barriers surrounding game meat; (ii) to quantify perceptions of its healthiness, safety, sustainability, price, and origin; (iii) to test which sociodemographic characteristics are associated with consumption, willingness, product knowledge, and price perception; (iv) to identify exploratory respondent profiles using multiple correspondence analysis; and (v) to examine factors independently associated with previous game-meat consumption and willingness to try game meat among non-consumers.

Because the questionnaire did not include a validated multi-item attitudinal scale, the analysis emphasises association, group comparison, dimension reduction, and exploratory respondent profiling rather than latent-construct measurement, a design choice made explicit in the Methods Section. The results are intended to inform producers, retailers, hunting associations, and public-health and food-policy stakeholders seeking to understand the demand side of the Romanian game meat market.

Beyond providing the first structured survey-based evidence from Romania, the findings contribute to the broader understanding of consumer acceptance of game meat in emerging European markets and may support the development of hypotheses and future research concerning wildlife-derived food products.

## 2. Materials and Methods

### 2.1. Study Design and Participants

A cross-sectional consumer survey was conducted in Romania using a structured, self-administered questionnaire distributed online via Google Forms (Google LLC, Mountain View, CA, USA). Participation was voluntary and anonymous, and all respondents provided informed consent before beginning the questionnaire. Eligible participants were residents of Romania aged 18 years or older. Recruitment followed a non-probability convenience-sampling approach. The Google Forms questionnaire link was distributed electronically through university-associated networks, including staff and student networks from veterinary education centres in Timișoara, București, Cluj-Napoca, and Iași, and was further circulated through digital social and messaging networks. Data were collected between 13 May 2025 and 3 May 2026. Because the questionnaire link was disseminated through digital networks rather than to a defined sampling frame, the number of individuals who received or viewed the survey invitation was not available; therefore, a conventional response or completion rate could not be calculated. To reduce duplicate participation, Google Forms was configured to permit only one submission per account. A total of 643 complete questionnaire records were initially obtained. Following eligibility screening, three respondents who selected the age category “<18 years” were excluded, resulting in a final analytical sample of 640 respondents.

Given the non-probability convenience-sampling design, the findings should be interpreted as describing the surveyed sample rather than as nationally representative estimates for the Romanian population. No formal a priori power calculation was performed because the study was designed as an exploratory convenience survey; the achieved sample size was determined by recruitment feasibility during the predefined data-collection period. The resulting analytical respondents sample was considered adequate for the principal descriptive and exploratory analyses, while subgroup analyses were interpreted according to their available sample sizes. The sample characteristics are presented in Section 3.1. The implications of this sample structure are further addressed in Section 4.6: Limitations. The de-identified analytical dataset used for the statistical analyses is provided in the Supplementary Materials (Data S1). Reporting of the cross-sectional and internet-administered survey was additionally assessed using the STROBE and CHERRIES checklists, provided in the Supplementary Materials as Files S2 and S3.

### 2.2. Questionnaire Design

The questionnaire was originally developed and administered in Romanian (*Percepția consumatorilor asupra cărnii de vânat*). Its development was informed by a review of recent European literature on game-meat consumption and consumer perceptions, with relevant themes concerning product awareness, perceived characteristics, and barriers to consumption adapted to the objectives of the present study and the Romanian context [2,9]. Prior to data collection, the draft questionnaire was reviewed for content and face validity by colleagues with expertise in veterinary public health, food safety, and animal production. It was subsequently pilot-tested in a convenience sample of 20 consumers and non-consumers of game meat to assess question clarity, the appropriateness of response options, and the functionality of the questionnaire flow and conditional routing. Minor linguistic and layout adjustments were made before the formal survey launch.

Data coding and statistical analysis were conducted using the original Romanian-language responses. Following completion of data collection, the Romanian questionnaire was translated into academic English for reporting and inclusion in the Supplementary Materials. Translation was performed by bilingual members of the research team and was subsequently reviewed and reconciled by a researcher proficient in academic English to preserve the intended meaning and scientific and domain-specific terminology. Because the English version was not used for questionnaire administration or primary data coding, an independent forward–back translation procedure was not performed. The complete English version is provided in the Supplementary Materials (File S1).

The instrument comprised items covering nine core areas: (i) sociodemographic characteristics (sex, age, area of residence, education, monthly income, occupation, and personal connection to hunting); (ii) awareness of game meat and of specific species; (iii) sources of information; (iv) consumption history, frequency, product forms, settings, and acquisition channels; (v) perceptions and associated characteristics (e.g., healthiness, natural/ecological status, taste, preparation difficulty, and safety); (vi) agreement with a set of statements regarding availability, preparation, ethics, provenance, and sustainability; (vii) barriers to consumption; (viii) factors that would encourage consumption; and (ix) willingness to consume game meat in the future.

Several items were designed as multiple-response questions, allowing the selection of more than one option, while others were intended for specific respondent subgroups according to previous consumption experience. Where respondents selected more options than the stated maximum, all recorded selections were retained because there was no objective basis for deleting individual selections. Specifically, questions regarding consumption frequency, consumed species, product forms, settings, acquisition channels, and factors encouraging more frequent consumption were analysed among respondents who reported previous game-meat consumption and provided valid responses. Where respondents reporting no previous game-meat consumption nevertheless had responses recorded for consumption-dependent items, these logically inapplicable responses were excluded, and the corresponding analyses were restricted to respondents reporting previous consumption. Future willingness to consume was assessed among respondents reporting no previous consumption.

The barrier item (Q5.1), although formulated for respondents who did not consume game meat, was also completed by a subset of respondents reporting previous consumption. Because the questionnaire did not separately distinguish current, former, and occasional consumers, these responses were retained for an exploratory comparison according to previous consumption experience. 

During data cleaning, responses recorded under semantically identical categories were harmonised before analysis. For the multiple-response items on product forms and consumption settings, respondents selecting only the consolidated “all of the above” option were coded as selecting each constituent response category (*n* = 2 for product forms and *n* = 57 for consumption settings). Minor spelling variants referring to the same predefined response option were also merged; this affected one response in the hunting-connection variable and three responses in the hunters/friends acquisition category. These harmonisations did not create new response categories but standardised equivalent responses for analysis.

This survey structure resulted in different applicable denominators across conditional items. Consequently, the corresponding items were analysed within their relevant subsamples, with sample sizes reported explicitly in Section 3: Results. Finally, one free-text item querying “other” game types had a 72.7% non-completion rate; therefore, it was treated qualitatively and excluded from the quantitative analysis.

### 2.3. Variable Classification

Prior to analysis, variables were classified according to their measurement level to guide the selection of appropriate statistical methods. Nominal variables included sex, area of residence, occupation, connection to hunting, and other categorical characteristics. Ordinal variables comprised age group, education level, income band, price perception, importance of origin, and the single-item self-rated information score regarding game meat, measured on a 1–5 scale. Binary indicators were derived for each option of the multiple-response items and for the principal binary outcomes, including previous game-meat consumption and willingness to try game meat in the future. The self-rated information score was treated as ordinal for inferential group comparisons.

### 2.4. Statistical Analysis

All statistical analyses were performed in Python version 3.13 (Python Software Foundation, Wilmington, DE, USA) utilising its standard open-source modules for data analysis (pandas, scipy.stats, statsmodels, scikit-learn, prince, and scikit-posthocs). Two-sided tests were used throughout, with the significance level set at α = 0.05.

Categorical variables were summarised as frequencies and percentages, alongside 95% Wilson score confidence intervals, which perform robustly for proportions near the bounds and for small subsample sizes. The self-rated information was summarised using the mean, median, standard deviation, and a 95% confidence interval. Multiple-response items were reported as the percentage of respondents within the applicable subsample who selected each specific option.

Bivariate associations between categorical outcomes and sociodemographic predictors were tested using Pearson’s chi-square test. When more than 20% of expected cell counts were below five, the Fisher–Freeman–Halton exact test was used instead of relying on the asymptotic Pearson chi-square *p*-value, and the resulting exact *p*-value was used for inference and multiplicity adjustment. Effect sizes were quantified using bias-corrected Cramér’s V (interpreted as negligible < 0.1, weak 0.1–0.2, moderate 0.2–0.3, strong > 0.3) and, for 2 × 2 tables, the phi coefficient. To account for multiple testing across the 35 sociodemographic association analyses, *p*-values were adjusted using the Benjamini–Hochberg false-discovery-rate procedure.

Respondents who completed the barrier item (Q5.1) were stratified according to previous game-meat consumption reported in Q4.1. Because current consumption status was not separately assessed, comparisons involving respondents with previous consumption were treated as exploratory. Differences in individual barriers were assessed using Pearson’s χ^2^ tests, with φ coefficients as effect-size measures and Bonferroni adjustment for the six comparisons.

Differences in the self-rated information score across groups were assessed non-parametrically because the score was measured on a 1–5 ordinal scale. The Mann–Whitney U test was employed for two-level factors and the Kruskal–Wallis H test for multi-level factors. Where the omnibus test was significant, Dunn’s post hoc test with a Bonferroni correction was applied. Effect sizes were reported as the rank-biserial correlation for Mann–Whitney and epsilon squared *ϵ*^2^ for Kruskal–Wallis. To maintain adequate group sizes for inferential testing, sparse categories were collapsed: age was grouped as 18–24, 25–34, and ≥35 years, while the two smallest education categories (*n* = 2 and *n* = 5) were merged with the high-school category.

To summarise the multivariate structure of respondent engagement and perception, multiple correspondence analysis (MCA) was applied to indicators covering game-species awareness (Q2.1), information sources (Q2.2), knowledge of where to obtain game meat (Q2.3), self-rated information (Q2.4), importance of origin (Q2.5), perceived characteristics (Q3.1), price perception (Q3.2), and agreement statements (Q3.3). The complete list of active MCA variables and their corresponding categories/indicators is provided in Supplementary Materials (Table S2). Sociodemographic and consumption-dependent variables were not used as active MCA variables. The number of retained dimensions was guided by the Benzécri-adjusted inertia. Respondent coordinates on the first two dimensions were subsequently clustered using a *k*-means algorithm. The optimal number of clusters was determined by evaluating the silhouette coefficient across *k* = 2 to 6 in conjunction with interpretability, leading to the retention of a three-cluster solution (*k* = 3). The robustness of the three-profile solution was additionally examined using clustering based on additional MCA dimensions and repeated resampling, with similarity of cluster assignments assessed using the adjusted Rand index.

Two multivariable binary logistic regression analyses were conducted. The first examined factors associated with previous game-meat consumption in the full analytical sample (*n* = 640) and included sex, residence, age group, education, personal income, connection to hunting, and self-rated information. The second examined factors associated with willingness to try game meat among non-consumers with valid responses (*n* = 152) and included sex, residence, age group, personal income, hunting connection, and self-rated information. Education was not included in the willingness model because the *n* = 152 non-consumer subgroup contained very small numbers in several education categories, particularly at the lowest and postgraduate levels, which would have increased model dimensionality and produced unstable category-specific estimates. Education also showed no meaningful bivariate association with willingness in this subgroup. Occupation was not included in the multivariable models because several occupational categories contained relatively few respondents and the variable partially overlapped conceptually with hunting connection through the active-hunter category. Hunting connection was retained as the more directly relevant measure of exposure to hunting networks, while limiting unnecessary model dimensionality. For both multivariable analyses, personal income was coded as a binary variable (any personal income versus no personal income) to reduce model dimensionality and avoid unstable estimates from relatively small category-specific cell counts, particularly in the willingness model (*n* = 152). The original income categories were retained for the bivariate analyses where each sociodemographic characteristic was evaluated separately. In the first analysis, hunting connection was represented by separate categories for family/friends involved in hunting and active hunter, with no hunting connection as the reference category; in the second analysis, hunting connection was dichotomised as any hunting connection versus none. Because self-rated information was measured on a five-point ordinal scale but entered as a one-point linear predictor in the logistic regression models, sensitivity analyses were performed by refitting each model with self-rated information specified as a categorical variable. The categorical and linear-score specifications were compared using likelihood-ratio tests. Multicollinearity was assessed using variance inflation factors (VIF). Model fit was evaluated using McFadden’s pseudo-*R*^2^ and the likelihood-ratio test, while calibration was assessed using the Hosmer–Lemeshow test. Results are reported as adjusted odds ratios (ORs) with corresponding 95% confidence intervals.

Because the questionnaire consisted primarily of single-item indicators, binary multiple-response options, and heterogeneous categorical variables rather than multi-item scales measuring common latent constructs, conventional psychometric procedures such as Cronbach’s alpha and exploratory or confirmatory factor analysis were not applicable. The analytical methods were therefore selected according to the categorical and ordinal structure of the data.

## 3. Results

### 3.1. Sample Characteristics

A total of 643 complete questionnaire records were initially available. Three respondents were excluded because they selected the age category “<18 years” and therefore did not meet the predefined eligibility criterion. The final analytical sample comprised 640 respondents (Table 1).

The respondents were predominantly female (60.3%, 95% CI [56.5–64.0]) and urban (62.7% [58.8, 66.3]). The age distribution was concentrated in the youngest bands, with 53.4% aged 18–24 and 24.5% aged 25–34. Educational attainment was comparatively high: 64.2% reported higher education and 7.8% postgraduate education. Students constituted the largest occupational group (57.5%), followed by respondents employed in fields other than the food sector (26.1%). Approximately one-third reported no personal income (33.0%). Most respondents reported no direct connection to hunting (61.6%), whereas 28.0% had family members or friends who were hunters and 10.5% identified themselves as active hunters.

### 3.2. Awareness, Information Sources, and Perceptions

**Figure 1:**
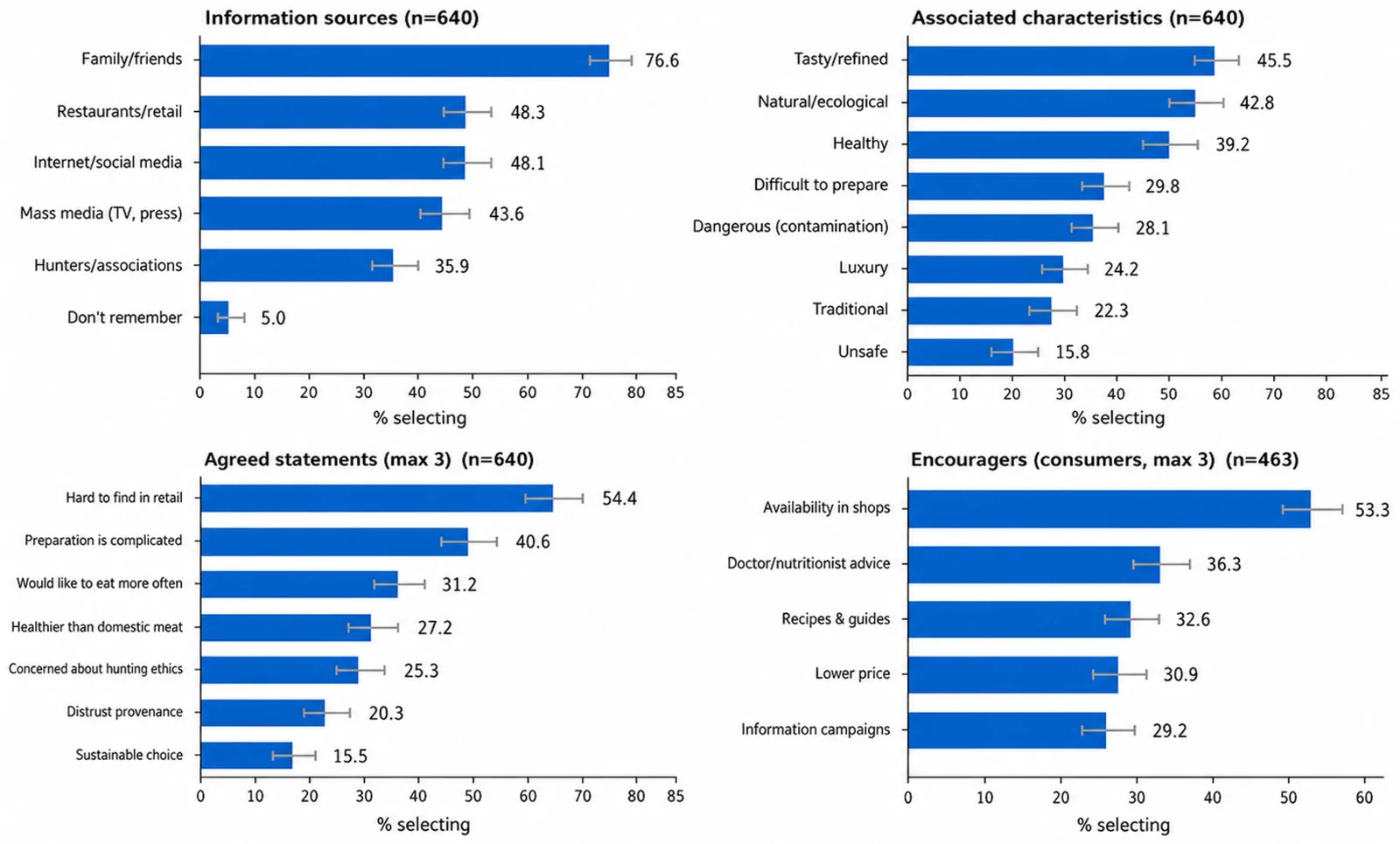
Multiple-response items summarising information sources, perceived characteristics, agreement statements, and factors that could encourage more frequent game-meat consumption. Bars show the percentage of respondents within the applicable analytical subsample selecting each option; whiskers represent 95% Wilson confidence intervals.

Awareness of individual game species was high and dominated by wild boar, recognised by 98.0% of respondents [96.6–98.8], followed by roe deer (91.1%), pheasant (89.5%), and red deer (88.9%) (Table 2, Figure 1). Personal networks were the most frequently reported source of information about game meat, with 76.6% selecting family or friends. Restaurants or retail outlets (48.3%) and the internet or social media (48.1%) were reported by approximately half of the sample, followed by mass media (43.6%) and hunters or hunting associations (35.9%). Respondents associated game meat with both favourable and unfavourable characteristics. The most frequently selected positive attributes were tasty/refined (45.5%), natural/ecological (42.8%), and healthy (39.2%). At the same time, 29.8% considered it difficult to prepare, 28.1% associated it with possible contamination, and 15.8% regarded it as unsafe.

Among the agreement statements, 54.4% considered game meat difficult to find in retail and 40.6% considered its preparation complicated. Almost one-third (31.2%) indicated that they would like to consume game meat more often. Smaller proportions agreed that game meat was healthier than meat from domestic animals (27.2%) or represented a sustainable food choice (15.5%), while 25.3% expressed concern about hunting ethics and 20.3% reported distrust regarding provenance.

On the single-response items (Table 3), the origin of meat was considered important or very important by 82.3% of respondents. Price was most commonly perceived as moderate (51.1%), whereas 33.3% considered game meat expensive and 15.6% considered it affordable. Only one-third of respondents (33.1%) reported knowing where game meat or game products could be purchased locally; 52.3% did not know and 14.5% were not interested. Self-rated information about game meat was relatively low overall. The mean score was 2.59 on the 1–5 scale (95% CI [2.49–2.69]), with a median of 2 and a standard deviation of 1.28. Overall, 54.2% of respondents rated their own level of information as either 1 or 2.

### 3.3. Consumption and Purchasing Behaviour

Of the 640 eligible respondents, 485 (75.8%, 95% CI [72.3-78.9]) reported having consumed game meat at least once, whereas 155 (24.2%) reported no previous consumption.

Among the 479 previous consumers providing a valid response on frequency, consumption was predominantly infrequent. More than one-third (37.8%) reported consuming game meat only occasionally, 21.3% consumed it once a year or less, and 26.7% a few times per year. Monthly consumption was reported by 11.9%, while weekly consumption was uncommon (2.3%).

Wild boar was the most frequently reported species among previous consumers (87.2%), followed by roe deer (62.9%), pheasant (55.9%), and red deer (47.2%). Among the 482 respondents providing information on product form, semi-prepared game products were reported by 68.9%, fresh meat by 66.0%, and prepared or processed meat by 63.9%.

Game meat was most commonly consumed at home (67.4%) or at the homes of friends or relatives (65.6%), whereas restaurants (38.4%) and events (35.7%) were less frequently reported settings. Acquisition was strongly linked to personal hunting networks: among the 472 respondents providing acquisition-channel information, 80.7% reported obtaining game meat directly from hunters or acquaintances. Restaurants were reported by 26.1%, specialised shops by 20.1%, and traditional markets by 10.8%.

Among 463 previous consumers who answered the item concerning factors that could encourage more frequent consumption, improved availability in shops or markets was the most frequently selected factor (53.3%). This was followed by recommendations from doctors or nutritionists (36.3%), access to recipes or preparation guides (32.6%), lower prices (30.9%), and information campaigns (29.2%).

Among the 155 respondents who reported never having consumed game meat, 152 answered the future-willingness item. Exactly half (76/152, 50.0%, 95% CI [42.1–57.9]) indicated that they would be willing to try game meat in the future.

### 3.4. Associations Between Sociodemographic Factors and Outcomes

Associations between sociodemographic characteristics and the five categorical outcomes are presented in Table 4. Effect sizes were considered alongside statistical significance, and Benjamini–Hochberg false-discovery-rate (FDR) correction was applied across the 35 comparisons.

**Figure 2:**
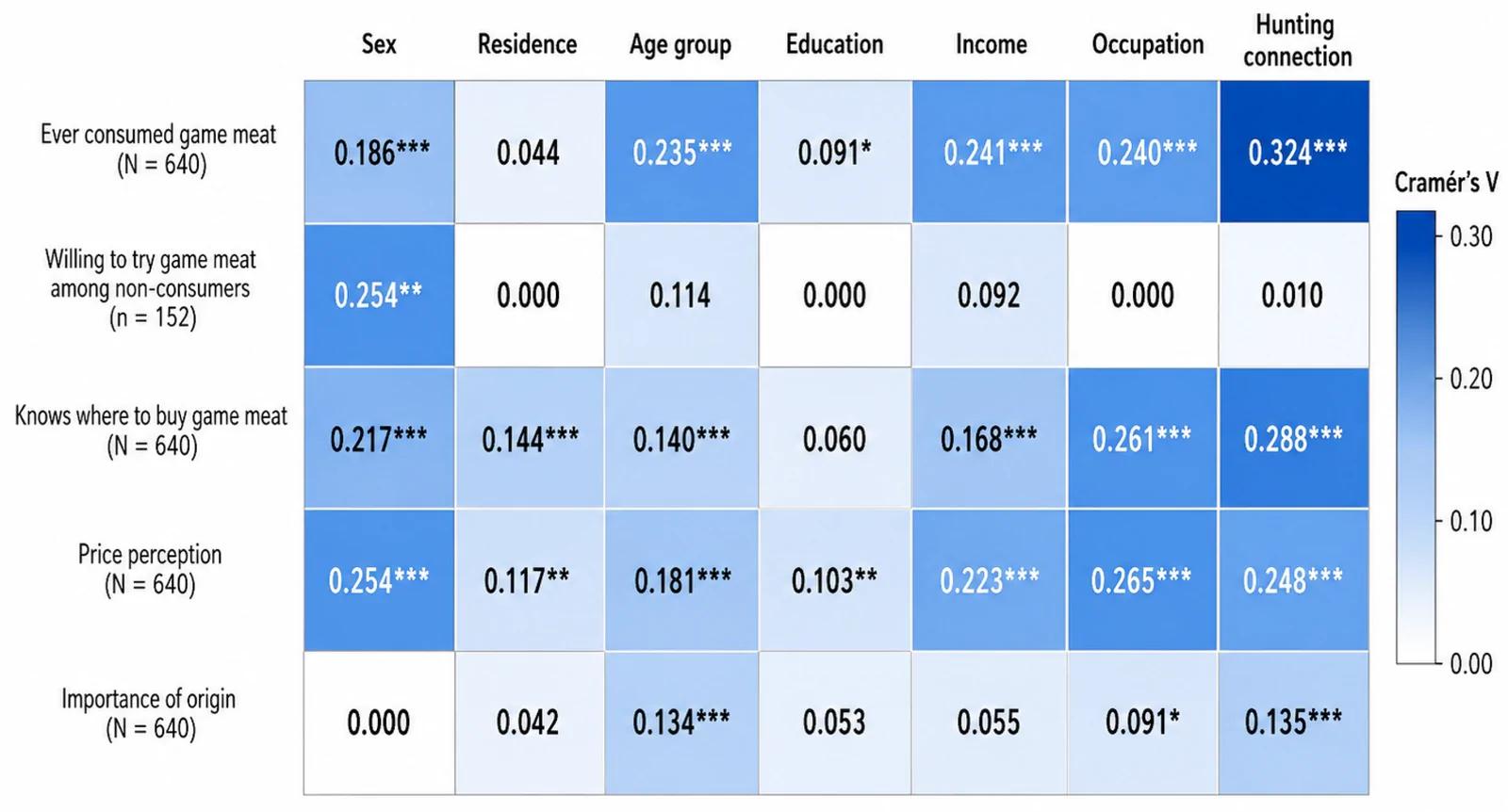
Heatmap of bias-corrected Cramér’s V values for associations between selected outcomes and sociodemographic characteristics. Darker colours indicate stronger associations. Analyses used *n* = 640, except willingness to try game meat (*n* = 152 non-consumers). Asterisks indicate Benjamini–Hochberg FDR-adjusted significance: q < 0.05 (*), q < 0.01 (**), and q < 0.001 (***).

For previous game-meat consumption, the largest association was observed for connection to hunting (Cramér’s V = 0.324), followed by income (V = 0.241), occupation (V = 0.240), and age group (V = 0.235). Connection to hunting therefore represented the only association reaching the predefined strong-effect threshold. Sex showed a weaker association (V = 0.186), whereas residence was not associated with previous consumption. Education reached statistical significance after FDR correction but showed a negligible effect size (V = 0.091), indicating limited practical differentiation between educational groups (Figure 2).

Among the 152 non-consumers who provided a valid response concerning future willingness, sex was the only sociodemographic characteristic associated with willingness after FDR correction (V = 0.254, FDR-adjusted *p* = 0.002). In this subgroup, 74.3% of male non-consumers (26/35) reported willingness to try game meat compared with 42.7% of female non-consumers (50/117). Residence, age, education, income, occupation, and connection to hunting were not significantly associated with willingness (Figure 2).

Knowledge of where game meat could be purchased locally was most strongly associated with hunting connection (V = 0.288) and occupation (V = 0.261), both moderate effects. Sex also showed a moderate association (V = 0.217), while associations with income, residence, and age were weak. Educational attainment was not significantly associated with knowledge of where to purchase game meat after FDR correction (Figure 2).

Perceived price was associated with all seven sociodemographic characteristics after multiplicity correction. The largest effects were observed for occupation (V = 0.265), sex (V = 0.254), hunting connection (V = 0.248), and income (V = 0.223), all within the moderate range. Associations with age, residence, and education were weaker (Figure 2).

Importance attributed to meat origin showed comparatively little sociodemographic differentiation. Age (V = 0.134) and hunting connection (V = 0.135) showed weak associations, while occupation reached statistical significance despite a negligible effect size (V = 0.091). Sex, residence, education, and income were not significantly associated with the importance attributed to origin after FDR correction (Figure 2).

### 3.5. Group Differences in Self-Rated Information

**Figure 3:**
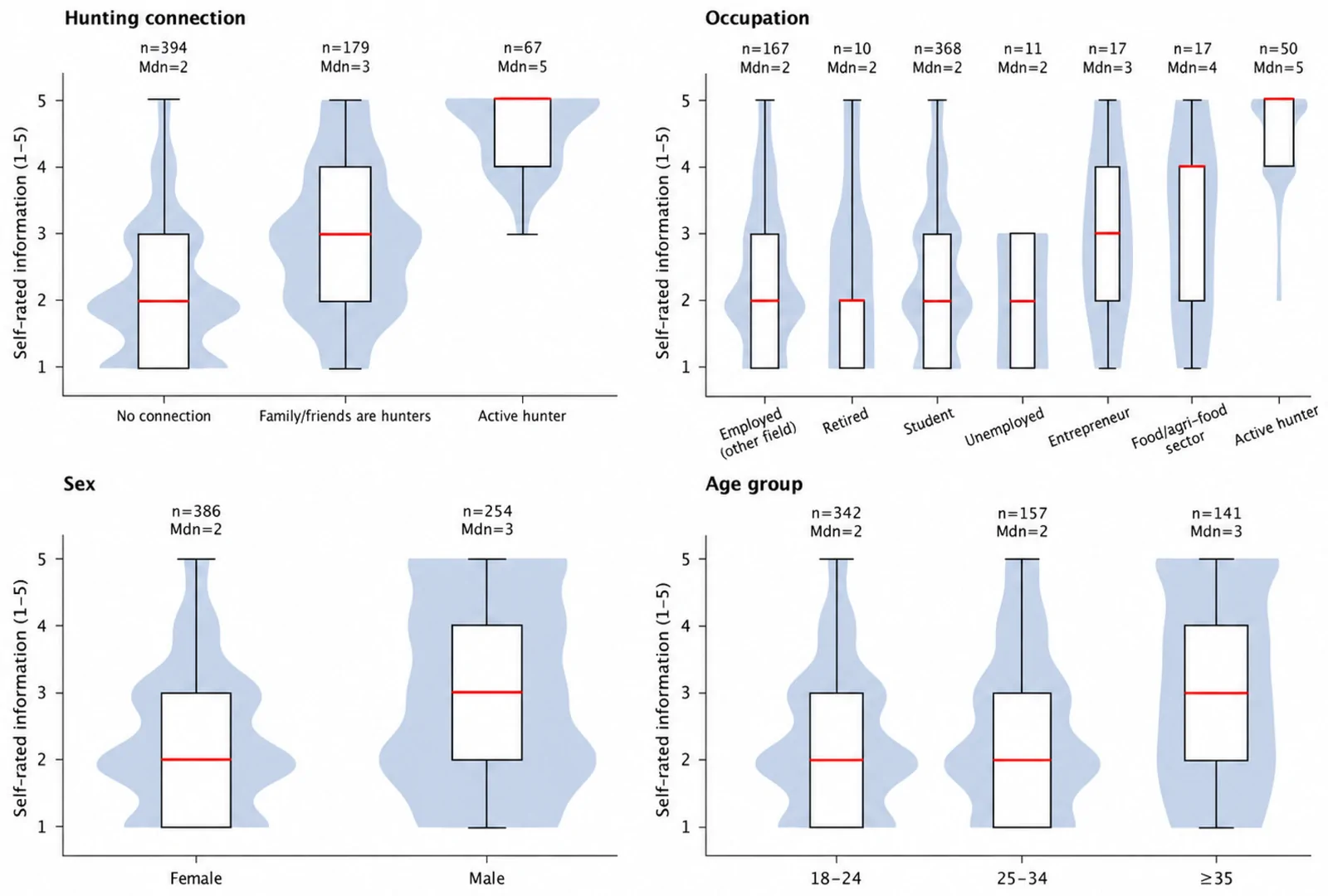
Distribution of self-rated information about game meat (1–5 scale) across selected sociodemographic groups. Violin plots show response distributions and boxplots indicate the interquartile range and median. Sample size (*n*) and median (Mdn) are shown above each group. Age was grouped as 18–24, 25–34, and ≥35 years for inferential analysis. Group differences were assessed using Mann–Whitney U or Kruskal–Wallis tests, as appropriate.

Self-rated information about game meat differed across sociodemographic groups (Table 5, Figure 3), with the clearest differences associated with hunting connection and occupation. Active hunters reported the highest levels of information, while men rated themselves as more informed than women and rural respondents more informed than urban respondents. Age and income showed smaller differences, whereas education had only a weak association with self-rated information. Post hoc comparisons showed a clear gradient across hunting-connection groups, with active hunters reporting higher information than respondents connected to hunters through family or friends, who in turn reported higher information than those without a hunting connection; age groups also differed significantly, whereas no pairwise education comparison remained significant after adjustment. The complete Dunn’s post hoc pairwise comparisons, including test statistics and Bonferroni-adjusted *p*-values, are provided in Supplementary Materials Table S1.

### 3.6. Exploratory Respondent Profiles Based on MCA and Clustering

**Figure 4:**
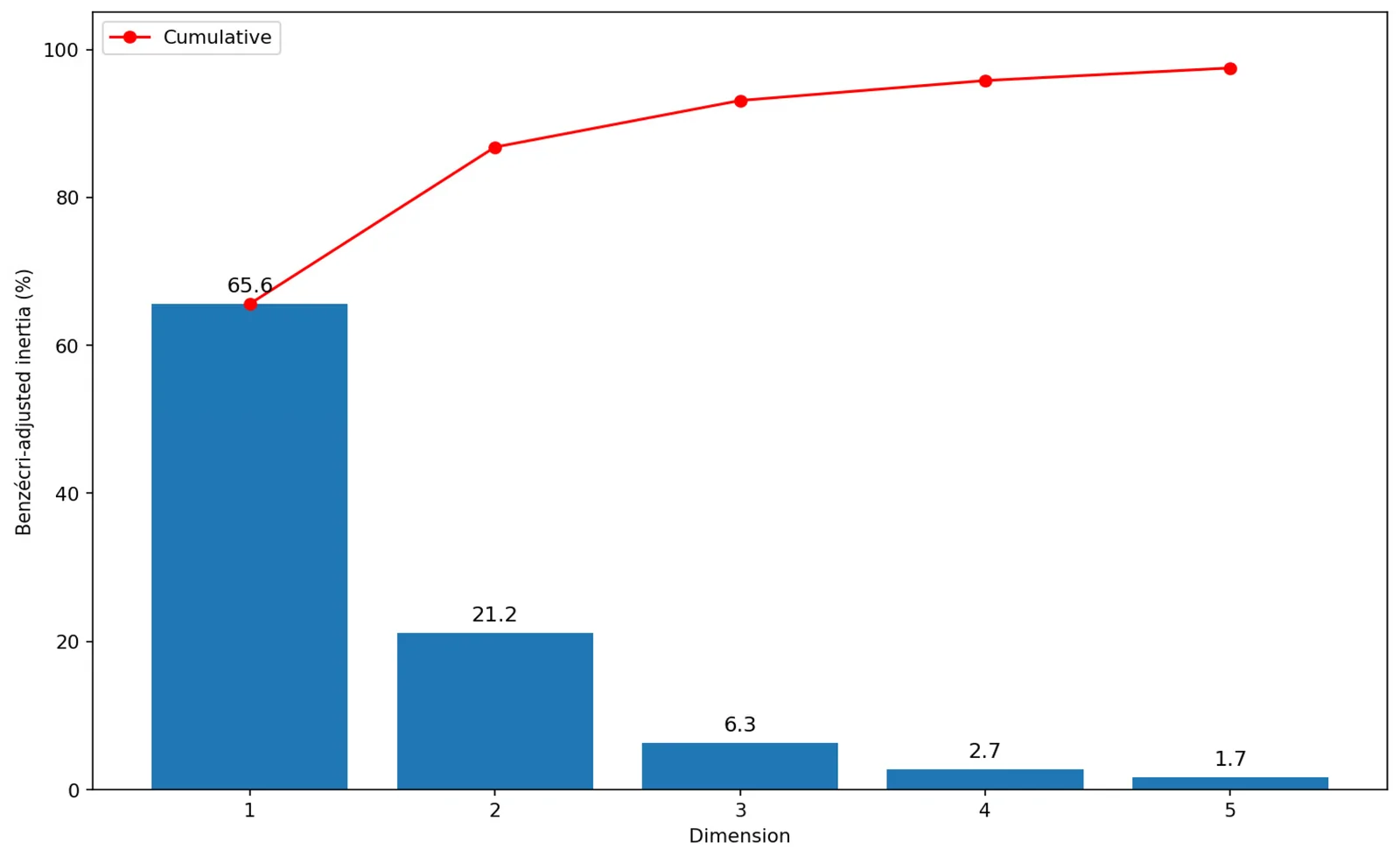
Scree plot of Benzécri-adjusted inertia from the multiple correspondence analysis (MCA). Bars show the adjusted inertia contributed by each of the first five MCA dimensions, while the red line represents cumulative adjusted inertia. Dimensions 1 and 2 accounted for 65.6% and 21.2% of the adjusted inertia, respectively, corresponding to 86.8% cumulatively. The first five dimensions together accounted for 97.5% of the adjusted inertia.

**Figure 5:**
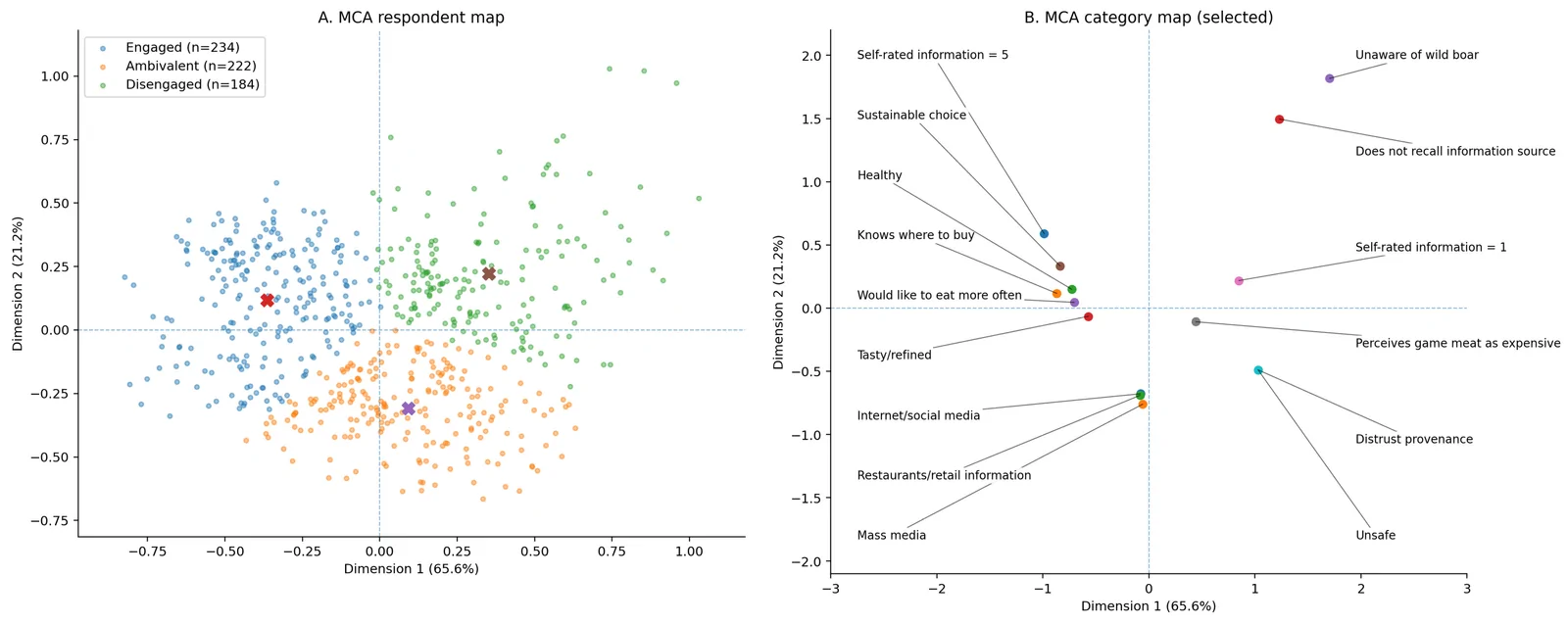
Multiple correspondence analysis (MCA) of the first two dimensions, explaining 86.8% of the Benzécri-adjusted inertia. (**A**) Respondent map showing the three exploratory profiles identified by k-means clustering: Engaged (*n* = 234), Ambivalent (*n* = 222), and Disengaged (*n* = 184); larger X symbols indicate cluster centroids. (**B**) Selected MCA category map for the *n* = 640 analytical sample. Dimension 1 (65.6%) mainly reflects engagement, familiarity, and perceptions of game meat, whereas Dimension 2 (21.2%) reflects information-source exposure and general awareness. Dashed lines indicate the origin.

Multiple correspondence analysis (MCA) provided a low-dimensional representation of the knowledge, awareness, and perception indicators. The first two dimensions accounted for 65.6% and 21.2% of the Benzécri-adjusted inertia, respectively, corresponding to 86.8% in total (Figure 4). Dimension 1 primarily reflected a gradient of engagement and familiarity with game meat, whereas Dimension 2 was more closely related to general awareness and exposure to information sources (Figure 5). These dimensions were therefore retained for the primary clustering analysis.

Among candidate solutions from k = 2 to k = 6, the three-cluster solution had the highest silhouette coefficient (0.401; k = 2: 0.372, k = 4: 0.374, k = 5: 0.381, k = 6: 0.372). The resulting groups were therefore retained as exploratory respondent profiles, rather than interpreted as definitive consumer segments. Sensitivity analyses using additional MCA dimensions produced highly similar cluster assignments (adjusted Rand index, 0.967–0.987), and resampling analysis also indicated good stability of the three-profile solution (mean adjusted Rand index 0.976).

**Figure 6:**
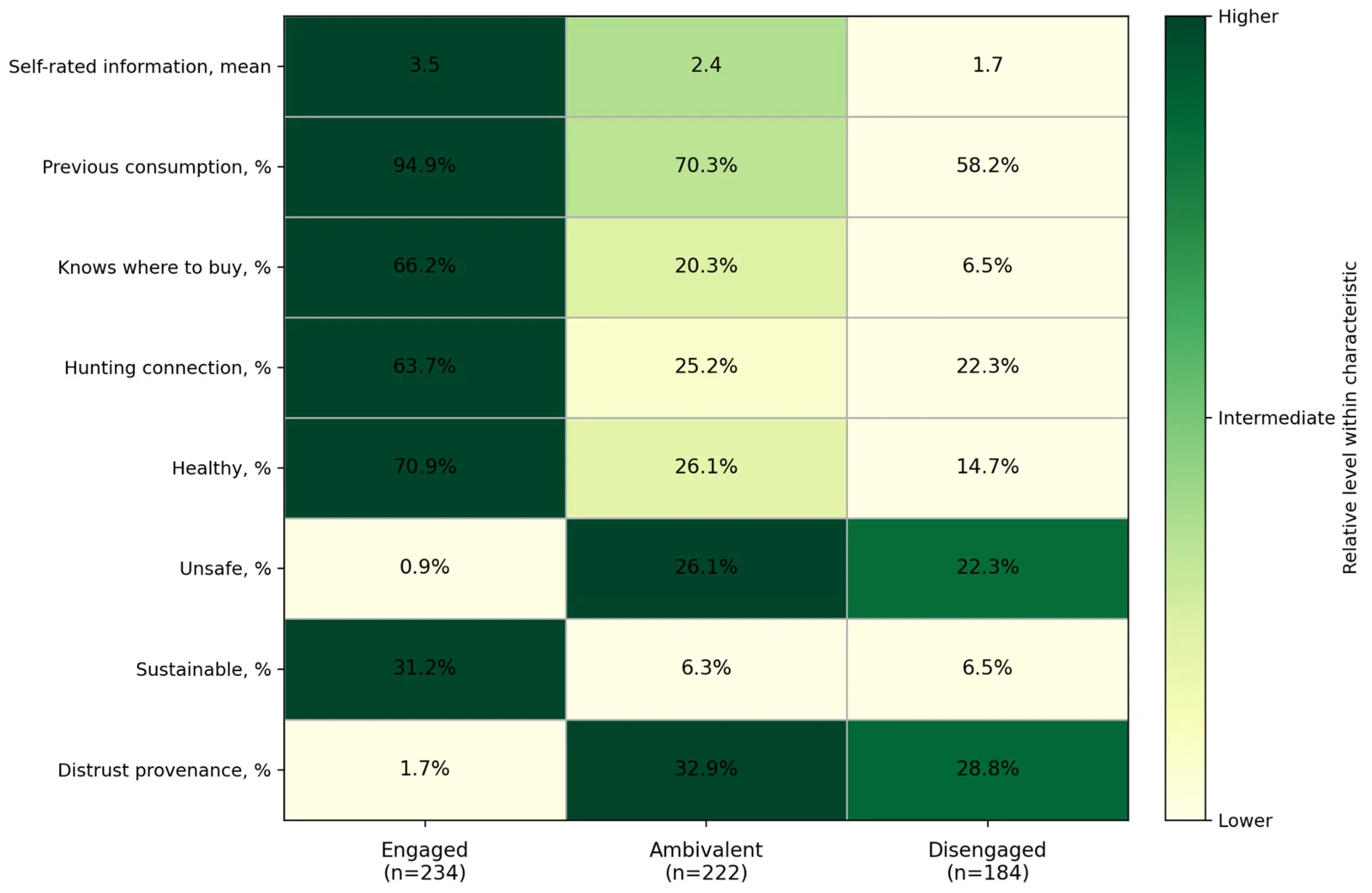
Heatmap of the three exploratory respondent profiles identified by MCA and k-means clustering: Engaged (*n* = 234), Ambivalent (*n* = 222), and Disengaged (*n* = 184). Cell values represent the mean self-rated information score or the percentage of respondents within each profile. Colour intensity indicates the relative level of each characteristic across profiles, including negatively framed characteristics such as unsafe perceptions and provenance distrust.

The three exploratory profiles comprised an Engaged group (*n* = 234), an Ambivalent group (*n* = 222), and a Disengaged group (*n* = 184) (Table 6, Figure 6). The profiles followed a general gradient in self-rated information, previous consumption, knowledge of where to buy game meat, and hunting connection. The Engaged profile showed the greatest familiarity and involvement with game meat, the Ambivalent profile occupied an intermediate position overall but showed greater uncertainty regarding safety and provenance, and the Disengaged profile showed the lowest overall familiarity and involvement. Because six of the characteristics shown in Table 6 were derived from active MCA variables, differences in these characteristics are partly expected from the clustering procedure and should not be interpreted as independent validation of the profiles. Given the exploratory nature of the clustering, these profiles should be regarded as descriptive patterns within the surveyed sample rather than as validated consumer typologies or evidence of a distinct market structure.

### 3.7. Factors Associated with Consumption and Willingness

**Figure 7:**
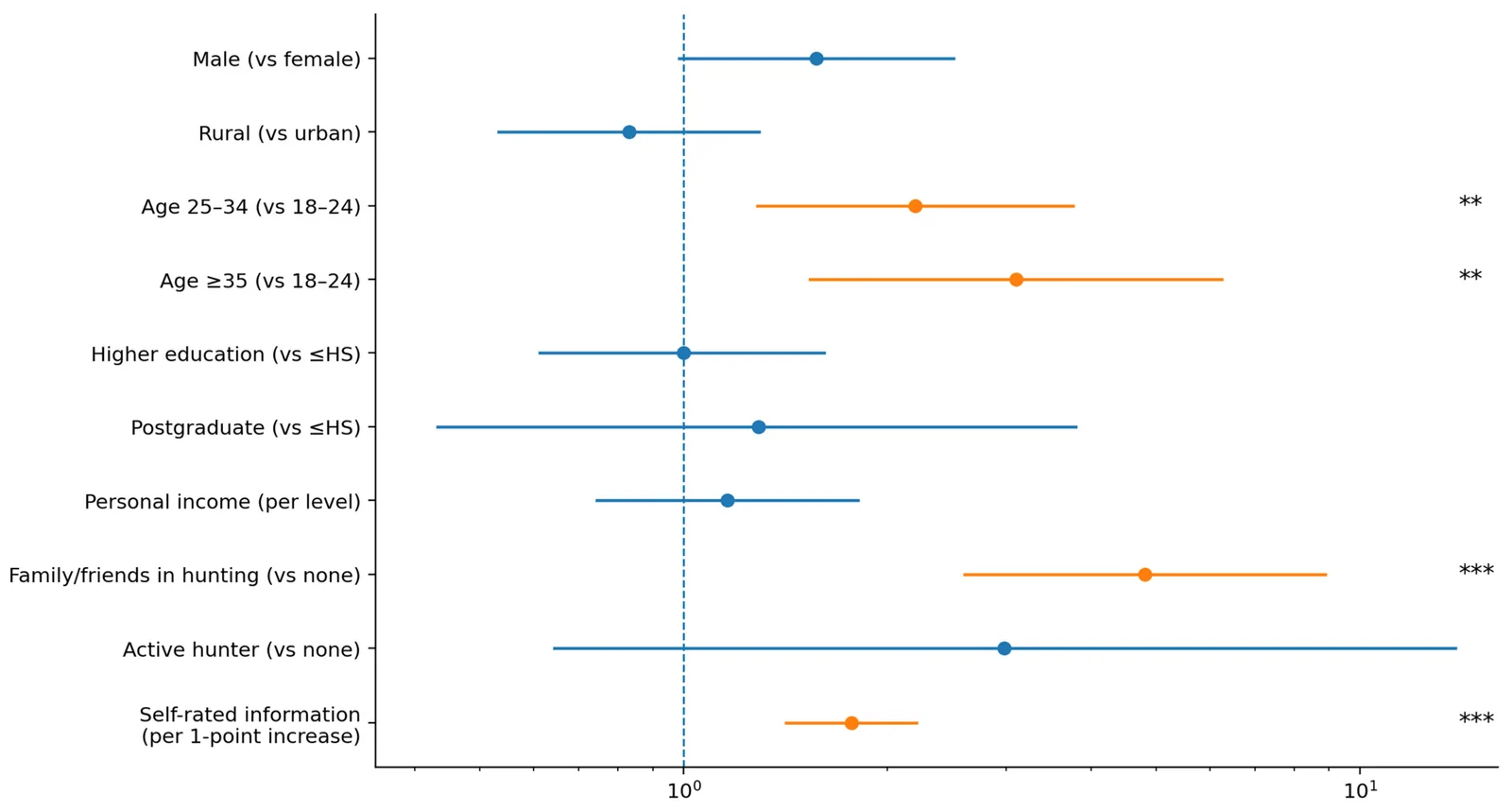
Forest plot of adjusted odds ratios (ORs) and 95% confidence intervals (CIs) for factors associated with previous game-meat consumption (*n* = 640). The dashed line indicates OR = 1. Significant associations are marked as ** *p* < 0.01 and *** *p* < 0.001.

In the multivariable logistic regression for previous game-meat consumption (Table 7, Panel A; Figure 7), the strongest adjusted association was observed among respondents with family members or friends involved in hunting, who had substantially higher odds of previous consumption than respondents without a hunting connection (OR = 4.81, 95% CI 2.59–8.94, *p* < 0.001). Higher odds were also observed among respondents aged 25–34 years (OR = 2.20, 95% CI 1.28–3.78, *p* = 0.004) and ≥35 years (OR = 3.10, 95% CI 1.53–6.28, *p* = 0.002), relative to those aged 18–24 years. Each one-point increase in self-rated information was associated with higher odds of previous consumption (OR = 1.77, 95% CI 1.41–2.22, *p* < 0.001). Sex, residence, education, personal income, and active-hunter status were not independently associated with previous consumption after adjustment. The model showed moderate explanatory power (McFadden pseudo-*R*^2^ = 0.205), with an overall significant likelihood-ratio test (*p* < 0.001) and no indication of poor calibration from the Hosmer–Lemeshow test (*p* = 0.384).

Among non-consumers with valid responses (*n* = 152), the model examining willingness to try game meat had substantially lower explanatory power (Table 7, Panel B). Male respondents had higher adjusted odds of willingness to try game meat than female respondents (OR = 3.59, 95% CI 1.52–8.48, *p* = 0.003). No independent associations were observed for residence, age, personal income, hunting connection, or self-rated information. The model had a McFadden pseudo-*R*^2^ of 0.068, indicating that the measured variables explained relatively little variation in willingness, although the overall likelihood-ratio test was significant (*p* = 0.045). The Hosmer–Lemeshow test did not indicate poor calibration (*p* = 0.988). Sensitivity analyses treating self-rated information as a categorical rather than linear predictor did not significantly improve model fit for either previous consumption (likelihood-ratio χ^2^ = 1.31, df = 3, *p* = 0.727) or willingness to try game meat (χ^2^ = 4.20, df = 3, *p* = 0.240). The more parsimonious linear-score specification was therefore retained in the primary models. The complete comparison between the linear-score and categorical specifications is provided in Supplementary Materials (Table S3).

### 3.8. Barriers to Game-Meat Consumption

Among respondents who completed the barrier item (Q5.1; *n* = 367), 152 had never consumed game meat, whereas 215 reported previous game-meat consumption. Because the questionnaire did not distinguish current, former, and occasional consumers, responses from the latter group were interpreted as reported barriers to consumption rather than specifically as reasons for limiting intake. Comparisons between the two groups were therefore considered exploratory (Table 8).

Respondents with no previous consumption most frequently reported lack of opportunity (63.2%), dislike of the idea of eating wild animals (43.4%), and ethical concerns regarding hunting (34.2%). Among respondents with previous consumption who completed the barrier item, the most frequently reported barriers were lack of opportunity (37.2%), difficulty with preparation (33.0%), and perceived expense (26.5%). Lack of opportunity and dislike of the idea of eating wild animals were significantly more frequent among respondents with no previous consumption, whereas difficulty with preparation was more frequent among those with previous consumption. Ethical concerns regarding hunting and perceived expense did not remain significant after correction for multiple comparisons, and contamination concerns did not differ between the groups.

## 4. Discussion

This survey provides a structured description of attitudes, knowledge, and behaviour toward game meat among respondents in Romania. Four findings stand out: previous consumption was common but generally infrequent; acquisition was strongly linked to personal and hunting networks rather than formal retail channels; perceptions combined favourable attributes with practical and safety reservations; and exploratory MCA and clustering identified three respondent profiles differing in familiarity, previous consumption, and perceptions of game meat.

### 4.1. Consumption Is Widespread but Occasional and Strongly Linked to Personal Networks

The high ever-consumption rate (75.8%) coexists with very low regular consumption (2.3% weekly) and heavy reliance on informal supply; 80.7% of respondents obtained game meat directly from hunters or acquaintances rather than from shops or markets. This mirrors the European pattern in which game meat functions as an occasional, socially embedded food rather than a routine retail purchase [2,9]. Comparable consumption patterns have also been reported outside Europe, where game meat remains primarily associated with cultural traditions, recreational hunting, and limited commercial distribution rather than routine household purchasing [18]. It is consistent with Polish findings that consumption is infrequent and often mediated by personal contact with hunters [9], and with Italian evidence that limited retail presence constrains demand for wild boar [5]. The dominance of wild boar in both awareness (98.0%) and consumption (87.2%) also parallels its prominence in Italian and Central European markets [5]. The practical implication is that the ceiling on Romanian game meat consumption is set less by rejection than by access: most people have tried it, but few can readily buy it.

This observation is consistent with broader European evidence indicating that market accessibility represents one of the principal determinants of regular game meat consumption. Even consumers who express favourable attitudes towards game meat often encounter practical constraints related to limited retail distribution, seasonal availability, and fragmented supply chains, reducing opportunities for repeated purchase [2,4,5,9]. Improving the visibility of game meat within formal retail networks may therefore be as important as increasing consumer awareness, since repeated exposure has been shown to reinforce purchasing habits and reduce uncertainty associated with less familiar food products [19].

### 4.2. A Positive but Qualified Product Image

Romanian respondents most often associated game meat with positive attributes—tasty/refined (45.5%), natural/ecological (42.8%), and healthy (39.2%)—echoing the “natural, lean, environmentally friendly” framing reported across Europe [2,7]. Yet these positives were tempered by concerns about difficult preparation (29.8%), contamination (28.1%), and outright safety (15.8%), and by widespread agreement that game meat is hard to find (54.4%) and complicated to cook (40.6%). The coexistence of a favourable image with practical and safety reservations is a recurring theme in the literature [2,9], and helps explain why positive attitudes do not translate into frequent consumption. The high importance attributed to provenance, 82.3% rated origin important or very important, indicates that transparent sourcing information is likely to be well received, consistent with the emphasis on traceability and provenance in recent European work [5,7].

The favourable perception of game meat as a natural and healthy food is supported by numerous compositional studies demonstrating its high protein content, favourable amino acid profile, lower intramuscular fat concentration, and comparatively higher proportions of polyunsaturated fatty acids than conventional livestock meat [18]. Nevertheless, positive nutritional perceptions alone rarely guarantee frequent consumption, as practical concerns regarding preparation methods, culinary familiarity, and confidence in food safety frequently outweigh nutritional considerations during purchasing decisions [20].

Notably, only 15.5% of respondents explicitly endorsed game meat as a sustainable choice. This relatively low proportion suggests that sustainability may not be a particularly salient attribute for many respondents, despite the sustainability framing frequently encountered in the scientific literature. Moreover, the environmental sustainability of wild game should not be regarded as universal, as it depends on the ecological context and on responsible wildlife management, harvesting, processing, and distribution practices.

The discrepancy between favourable perceptions and relatively infrequent consumption may also reflect the well-described attitude–behaviour gap observed in food choice research. Consumers frequently express positive attitudes towards products perceived as healthy or sustainable, yet purchasing decisions remain constrained by convenience, availability, habits, perceived risk, and culinary confidence [21].

### 4.3. Hunting Connection and Familiarity Are Associated with Game-Meat Consumption

Connection to hunting showed the strongest bivariate association with previous game-meat consumption (Cramér’s V = 0.324), and having family members or friends involved in hunting showed the strongest adjusted association in the multivariable analysis (OR = 4.81, 95% CI [2.59–8.94]). Self-rated information also differed more strongly according to hunting connection (ε^2^ = 0.286) and occupation (ε^2^ = 0.179) than according to education (ε^2^ = 0.007). Older age and higher self-rated information were also independently associated with higher odds of previous consumption. These findings are consistent with previous studies indicating that proximity to hunting and familiarity with game meat are important correlates of consumption [2,9,11]. However, the cross-sectional design does not establish directionality, particularly for self-rated information: previous consumption may itself increase familiarity and perceived knowledge of game meat. Together with the lack of an independent association with educational attainment, the findings suggest that practical exposure and familiarity may be more closely related to previous consumption than formal education alone.

From a behavioural perspective, repeated exposure to a food may increase familiarity and reduce the uncertainty associated with less familiar products. Individuals who have regular contact with hunters or game-meat consumers may consequently develop greater familiarity with flavour characteristics, preparation methods, and product sourcing. Similar mechanisms have been described for traditional, regional, and unfamiliar food products [22,23,24,25].

Interestingly, formal educational attainment showed little relationship with previous consumption after adjustment. Although education reached statistical significance in the bivariate analysis, its effect size was negligible, and it was not independently associated with previous consumption in the multivariable model. This suggests that practical experience and direct exposure may be more relevant to game-meat familiarity than educational attainment itself. Previous consumer research has similarly indicated that experiential exposure, tasting opportunities, and social interaction may contribute to acceptance of unfamiliar or underutilised foods [20,26,27,28].

Among non-consumers, the multivariable analysis of willingness to try game meat had substantially lower explanatory power (McFadden pseudo-*R*^2^ = 0.068), with male sex as the only independently associated variable. This indicates that the demographic and familiarity-related variables included in the model explained relatively little of the variation in willingness. Other factors not directly captured by the questionnaire, such as ethical orientation, sensory expectations, perceived risk, food neophobia, or attitudes toward hunting, may therefore warrant investigation in future studies.

### 4.4. Exploratory Respondent Profiles and Reported Barriers

The exploratory MCA and clustering analysis identified a clear gradient of engagement with game meat, broadly consistent with patterns described in previous consumer studies [11]. The “engaged” profile combined high self-rated information, high previous consumption, greater knowledge of where to obtain game meat, and minimal safety concern. The “ambivalent” profile showed intermediate levels of previous consumption and information together with the highest safety concern and provenance distrust; and the “disengaged” profile showed the lowest self-rated information, purchasing knowledge, and previous consumption. The analysis of barriers among non-consumers provided a complementary perspective. Lack of opportunity was the most frequently reported reason for not consuming game meat (63.2%), followed by discomfort with the idea of eating wild animals (43.4%) and the perception that hunting is unethical (34.2%). Fear of contamination (21.1%), uncertainty regarding preparation (19.7%), and perceived expense (17.1%) were reported less frequently. These findings suggest that non-consumption within this subgroup reflects a combination of limited exposure or access and personal or ethical reservations rather than a single dominant obstacle. Preparation uncertainty, although not among the leading barriers in the present non-consumer subgroup, has also been identified as a potentially modifiable constraint in previous European studies [2,9].

Respondents with previous consumption who also completed the barrier item reported lack of opportunity less frequently (37.2%) and preparation difficulty more frequently (33.0%) than respondents with no previous consumption. Because current, former, and occasional consumption could not be distinguished, these comparisons remain exploratory.

Exploratory respondent profiling can be useful in food-consumer research because individuals may differ in their familiarity, expectations, and concerns. Rather than treating the three profiles identified here as established market segments, future studies could evaluate whether differentiated communication approaches are appropriate for respondents with different levels of familiarity and engagement. For example, more engaged respondents may be particularly receptive to information concerning provenance and sustainability, whereas less familiar or more sceptical respondents may place greater emphasis on food safety and culinary preparation [29,30]. Similar differences in familiarity, perceived benefits, and concerns have been reported for alternative protein products [31,32]. These observations support further investigation of differentiated communication approaches within the game-meat sector.

### 4.5. Implications

Given the non-probability convenience sample and cross-sectional design, the following implications should be regarded as hypotheses and priorities for future evaluation rather than as interventions demonstrated by the present study to increase game-meat consumption.

For producers and retailers, the findings suggest that organised and visible retail availability and clear provenance labelling may represent relevant areas for future evaluation, particularly because limited access emerged as an important reported constraint. For hunting associations and food-sector actors, the provision of recipes, preparation guidance, and trusted safety information may address barriers related to culinary familiarity and food-safety concerns. For public-health and food-policy stakeholders, the high importance attributed to provenance and the reported interest in recommendations from doctors or nutritionists (36.3%) suggest that credible nutritional and safety communication may support consumer confidence, provided that it also acknowledges the specific safety considerations associated with wild-sourced meat. Because male non-consumers showed higher willingness to try game meat than female non-consumers, gender-related differences could also be explored in future communication research; however, this finding should be interpreted cautiously because sex was the only significant factor in a model with limited explanatory power. Retailers could additionally evaluate dedicated game-meat sections accompanied by clear information on species, origin, veterinary inspection, and recommended cooking practices. Such point-of-sale information may help reduce uncertainty among consumers unfamiliar with game meat while improving product visibility within conventional retail environments.

From a sustainability perspective, legally and responsibly harvested game meat may represent a complementary protein source within diversified food systems. However, its environmental benefits should not be assumed to be universal, as they depend on wildlife-management practices, harvesting intensity, biodiversity considerations, processing, transport, and distribution. Compared with intensive livestock systems, game meat may involve lower reliance on concentrated feeding systems, housing infrastructure, and routine veterinary inputs, although the overall sustainability of individual supply chains remains context dependent [2]. For policy makers, support for short supply chains, certified processing facilities, and transparent distribution systems could be evaluated as possible approaches to improving market accessibility while maintaining food-safety and traceability standards. Such measures may also provide opportunities for cooperation among consumers, hunting associations, processors, and rural communities.

The increasing interest in game meat may also be considered within a One Health perspective, which recognises the interconnectedness of wildlife, environmental health, animal health, and human health [33]. Appropriate surveillance of foodborne pathogens and antimicrobial resistance in wild ungulates, together with veterinary inspection and effective traceability systems, may be particularly relevant for maintaining consumer confidence and supporting the safe utilisation of wildlife-derived foods [10,17]. Collaboration among hunters, veterinarians, food business operators, and public-health authorities may further support consistent standards for carcass inspection, hygienic processing, and risk communication throughout the game-meat supply chain.

For the food industry, opportunities may exist to develop value-added game-meat products that improve convenience and reduce perceived preparation difficulty. Ready-to-cook products, standardised portion sizes, recipe-based packaging, and educational materials may help address some of the practical barriers reported by both previous consumers and potential consumers [2,29,34]. Partnerships among retailers, restaurants, culinary schools, and hunting organisations could also be evaluated as ways of increasing familiarity with game meat through tasting events, cooking demonstrations, or seasonal promotional activities. The effectiveness of such initiatives in changing consumer behaviour would, however, require direct evaluation.

As interest in alternative and sustainably produced animal proteins continues to develop across Europe, the game-meat sector in Romania may represent a relevant niche within the wider food market. Improvements in retail availability, traceability, consumer information, and product diversification may be particularly relevant to its future development, although their effects on consumer uptake cannot be established from the present cross-sectional survey.

Taken together, the findings suggest that broader access to game meat in Romania may depend not only on consumer attitudes but also on practical factors such as product accessibility, retail visibility, culinary familiarity, transparent communication, and confidence in food safety. These areas therefore represent potential priorities for future consumer research and intervention studies rather than measures demonstrated by the present study to increase consumption.

### 4.6. Limitations

Several limitations qualify these findings. First, the sample was recruited using non-probability convenience sampling and was predominantly young, highly educated, and student-dominated; therefore, it is not representative of the Romanian population, and the findings should be interpreted as describing the surveyed sample rather than providing national estimates. Recruitment was facilitated in part through academic and student networks associated with veterinary education centres, which may have contributed to the high proportion of students and highly educated respondents and may also have preferentially reached individuals with greater familiarity with animal-source foods, veterinary topics, or food-safety issues. This recruitment-channel effect may therefore have influenced some of the observed levels of awareness and familiarity with game meat. The relatively high proportion of respondents with hunting connections may also have contributed to the observed levels of familiarity with and previous consumption of game meat.

Second, the cross-sectional design allows identification of associations but not causal relationships. Accordingly, the regression models describe adjusted associations rather than causal effects. In particular, the association between self-rated information and previous consumption may reflect greater familiarity resulting from prior experience as well as any possible influence of information on consumption. Similarly, the strong association between having family members or friends involved in hunting and previous consumption cannot establish directionality, because individuals with a pre-existing interest in game meat may also be more likely to develop or maintain connections with hunting networks.

Third, all measures were self-reported and may be affected by recall and social-desirability bias. Self-rated information was assessed using a single ordinal item and should therefore be regarded as perceived familiarity rather than an objective measure of knowledge.

Fourth, the questionnaire relied mainly on single-item and multiple-response indicators rather than validated multi-item psychometric scales. Consequently, conventional assessment of internal consistency and construct validity was not applicable. The respondent profiles derived from MCA and clustering should therefore be interpreted as exploratory, data-driven patterns within this sample rather than as validated consumer segments, despite their good stability in sensitivity analyses.

Fifth, the questionnaire did not distinguish between current, former, and occasional game-meat consumers. This limits interpretation of the barrier item because some respondents who reported previous consumption also completed the question concerning reasons for not consuming game meat. Comparisons involving this subgroup were therefore treated as exploratory. The original questionnaire also omitted the 6001–7000 RON income interval, which represents an instrument-design limitation and should be corrected in future applications. Finally, the free-text “other game types” item was largely incomplete and was not analysed quantitatively.

Future research should employ probability-based or quota-based sampling to improve representativeness, use broader recruitment channels outside academic and veterinary networks, incorporate validated attitudinal and knowledge measures, distinguish current from former and occasional consumption, and consider longitudinal or choice-based designs to move beyond cross-sectional associations.

Despite these limitations, the relatively large sample and broad analytical approach provide a useful baseline for future studies of game-meat acceptance in Romania and for subsequent research using more representative sampling designs. 

## 5. Conclusions

In this cross-sectional online convenience sample of 640 respondents in Romania, game meat was widely recognised but generally consumed infrequently, with previous consumption strongly linked to personal hunting networks. Wild boar was the most widely recognised and most frequently consumed game species. Previous game-meat consumption was independently associated with older age, having family members or friends involved in hunting, and higher self-rated information, whereas formal education was not independently associated with consumption.

Exploratory MCA and clustering identified three respondent profiles reflecting different levels of familiarity, previous consumption, and perceptions of game meat. These profiles should be interpreted as descriptive patterns within the surveyed sample rather than as validated market segments.

Among respondents with no previous consumption, the main reported barriers were lack of opportunity, discomfort with the idea of eating wild animals, and ethical concerns regarding hunting. Respondents with previous consumption who also completed the barrier item more frequently reported difficulties related to preparation, although these comparisons were exploratory because current consumption status was not separately assessed.

Overall, the findings suggest that access, practical familiarity, information on provenance and safety, and culinary guidance may be relevant considerations for future consumer-oriented initiatives concerning game meat in Romania. Given the non-probability sampling design and cross-sectional nature of the study, these findings should be regarded as a basis for further research using more representative samples and longitudinal or choice-based approaches.

## Figures and Tables

**Table 1:** Sociodemographic profile of the sample (*n* = 640).

Characteristic	Category	*n*	%	95% CI
**Sex**	Female	386	60.3	[56.5–64.0]
	Male	254	39.7	[36.0–43.5]
**Age**	18–24	342	53.4	[49.6–57.3]
	25–34	157	24.5	[21.4–28.0]
	35–44	60	9.4	[7.4–11.9]
	45–54	48	7.5	[5.7–9.8]
	55–64	30	4.7	[3.3–6.6]
	65+	3	0.5	[0.2–1.4]
**Residence**	Urban	401	62.7	[58.8–66.3]
	Rural	239	37.3	[33.7–41.2]
**Education**	No formal education	2	0.3	[0.1–1.1]
	Primary/lower-secondary	5	0.8	[0.3, 1.8]
	High school	172	26.9	[23.6–30.4]
	Higher education	411	64.2	[60.4–67.8]
	Postgraduate	50	7.8	[6.0–10.2]
**Monthly income**	No personal income	211	33.0	[29.4–36.7]
	< 2000 RON	107	16.7	[14.0–19.8]
	2000–4000 RON	119	18.6	[15.8–21.8]
	4001–6000 RON	121	18.9	[16.1–22.1]
	>7000 RON	82	12.8	[10.4–15.6]
**Occupation**	Student	368	57.5	[53.6–61.3]
	Employed (other field)	167	26.1	[22.8–29.6]
	Active hunter (occupation)	50	7.8	[6.0–10.2]
	Food/agri-food sector	17	2.7	[1.7–4.2]
	Entrepreneur	17	2.7	[1.7–4.2]
	Unemployed	11	1.7	[1.0–3.1]
	Retired	10	1.6	[0.9–2.9]
**Connection to hunting**	No connection	394	61.6	[57.7–65.3]
	Family/friends hunters	179	28.0	[24.6–31.6]
	Active hunter (hunting connection)	67	10.5	[8.3–13.1]

Legend: *n*, number of respondents in each category; CI, confidence interval (95% Wilson score interval for proportions). The occupational category “Active hunter” refers to respondents reporting hunting as their principal occupation, whereas “Active hunter” under connection to hunting includes all respondents reporting active participation in hunting, irrespective of their principal occupation.

**Table 2:** Multiple-response items: percentage of respondents selecting each option.

Item	Option	*n*	%	95% CI
**Awareness of game types (*n* = 640)**	Wild boar	627	98.0	[96.6–98.8]
	Roe deer	583	91.1	[88.6–93.1]
	Pheasant	573	89.5	[86.9–91.7]
	Red deer	569	88.9	[86.2–91.1]
	Other	248	38.8	[35.1–42.6]
**Information sources (*n* = 640)**	Family/friends	490	76.6	[73.1–79.7]
	Restaurants/retail	309	48.3	[44.4–52.2]
	Internet/social media	308	48.1	[44.3–52.0]
	Mass media	279	43.6	[39.8–47.5]
	Hunters/hunting associations	230	35.9	[32.3–39.7]
	Does not remember	32	5.0	[3.6–7.0]
**Perceived characteristics (*n* = 640)**	Tasty/refined	291	45.5	[41.6–49.3]
	Natural/ecological	274	42.8	[39.0–46.7]
	Healthy	251	39.2	[35.5–43.1]
	Difficult to prepare	191	29.8	[26.4–33.5]
	Potentially contaminated/dangerous	180	28.1	[24.8–31.7]
	Luxury	155	24.2	[21.1–27.7]
	Traditional	143	22.3	[19.3–25.7]
	Unsafe	101	15.8	[13.2, 18.8]
**Agreement with statements (*n* = 640)**	Difficult to find in retail	348	54.4	[50.5–58.2]
	Preparation is complicated	260	40.6	[36.9–44.5]
	Would like to consume more often	200	31.2	[27.8–34.9]
	Healthier than domestic meat	174	27.2	[23.9–30.8]
	Concerned about hunting ethics	162	25.3	[22.1–28.8]
	Distrusts provenance	130	20.3	[17.4–23.6]
	Sustainable choice	99	15.5	[12.9–18.5]
**Game types consumed (*n* = 485)**	Wild boar	423	87.2	[83.9–89.9]
	Roe deer	305	62.9	[58.5–67.1]
	Pheasant	271	55.9	[51.4–60.2]
	Red deer	229	47.2	[42.8–51.7]
	Other	152	31.3	[27.4–35.6]
**Product forms consumed (*n* = 482)**	Semi-prepared products	332	68.9	[64.6–72.8]
	Fresh meat	318	66.0	[61.6–70.1]
	Prepared/processed meat	308	63.9	[59.5–68.1]
**Consumption settings (*n* = 482)**	Home	325	67.4	[63.1–71.5]
	Friends/relatives	316	65.6	[61.2–69.7]
	Restaurant	185	38.4	[34.1–42.8]
	Event	172	35.7	[31.5–40.1]
**Acquisition channels (*n* = 472)**	Hunters/friends	381	80.7	[76.9–84.0]
	Restaurants	123	26.1	[22.3–30.2]
	Specialised shops	95	20.1	[16.8–24.0]
	Traditional markets	51	10.8	[8.3–13.9]
**Factors encouraging consumption (*n* = 463)**	Better availability in shops/markets	247	53.3	[48.8–57.8]
	Recommendation from doctor/nutritionist	168	36.3	[32.0–40.8]
	Recipes/preparation guides	151	32.6	[28.5–37.0]
	Lower price	143	30.9	[26.8–35.2]
	Information campaigns	135	29.2	[25.2–33.5]

Legend: *n* indicates the number of respondents included for each item. Some items were assessed only in applicable respondent subgroups, and multiple responses were allowed; therefore, *n* may differ between items and percentages may sum to more than 100%. Values in brackets are 95% Wilson confidence intervals.

**Table 3:** Distribution of knowledge, perception, and behavioural variables.

Variable	Response	*n*	%	95% CI
**Knows where to buy game meat**	No	335	52.3	[48.5–56.2]
	Yes	212	33.1	[29.6–36.9]
	Not interested	93	14.5	[12.0–17.5]
**Importance of origin**	Indifferent	32	5.0	[3.6–7.0]
	Never thought about it	81	12.7	[10.3–15.5]
	Important	203	31.7	[28.2–35.4]
	Very important	324	50.6	[46.8–54.5]
**Price perception**	Affordable	100	15.6	[13.0–18.6]
	Moderate	327	51.1	[47.2–54.9]
	Expensive	213	33.3	[29.7–37.0]
**Ever consumed game meat**	Yes	485	75.8	[72.3–78.9]
	No	155	24.2	[21.1–27.7]
**Consumption frequency (n = 479)**	Only occasionally	181	37.8	[33.6–42.2]
	Once a year or less	102	21.3	[17.9–25.2]
	A few times a year	128	26.7	[23.0–30.9]
	Monthly	57	11.9	[9.3–15.1]
	Weekly	11	2.3	[1.3–4.1]
**Willing to try game meat in future** **(n = 152 non-consumers)**	Yes	76	50.0	[42.1–57.9]
	No	76	50.0	[42.1–57.9]

Legend: *n* indicates the number of respondents. Percentages for the first four variables are based on the full analytical sample (*n* = 640). Consumption frequency was calculated among previous consumers providing a valid frequency response (*n* = 479). Future willingness was calculated only among respondents who had never consumed game meat and provided a valid response to the willingness item (*n* = 152).

**Table 4:** Associations between sociodemographic characteristics and key categorical outcomes (chi-square tests with Cramér’s V).

Outcome	Predictor	χ^2^	df	*p*	FDR- Adjusted q	Cramér’s V	Effect
**Ever consumed game meat (*n* = 640)**	Sex	23.16	1	<0.001	<0.001	0.186	Weak
	Residence	2.26	1	0.133	0.177	0.044	Negligible
	Age group	37.19	2	<0.001	<0.001	0.235	Moderate
	Education	7.30	2	0.026	0.040	0.091	Negligible
	Income	40.97	4	<0.001	<0.001	0.241	Moderate
	Occupation ^a^	42.92	6	<0.001	<0.001	0.240	Moderate
	Hunting connection	68.95	2	<0.001	<0.001	0.324	Strong
**Willing to try game meat among non-consumers (*n* = 152)**	Sex	10.73	1	0.001	0.002	0.254	Moderate
	Residence	0.00	1	1.000	1.000	0.000	Negligible
	Age group	3.96	2	0.138	0.177	0.114	Weak
	Education ^a^	0.23	2	1.000	1.000	0.000	Negligible
	Income	5.32	4	0.256	0.299	0.092	Negligible
	Occupation ^a^	3.67	5	0.712	0.755	0.000	Negligible
	Hunting connection ^a^	2.03	2	0.577	0.652	0.010	Negligible
**Knows where to buy game meat (*n* = 640)**	Sex	31.97	2	<0.001	<0.001	0.217	Moderate
	Residence	15.30	2	<0.001	<0.001	0.144	Weak
	Age group	29.05	4	<0.001	<0.001	0.140	Weak
	Education	8.57	4	0.073	0.106	0.060	Negligible
	Income	43.90	8	<0.001	<0.001	0.168	Weak
	Occupation ^a^	99.14	12	<0.001	<0.001	0.261	Moderate
	Hunting connection	110.16	4	<0.001	<0.001	0.288	Moderate
**Price perception (*n* = 640)**	Sex	43.34	2	<0.001	<0.001	0.254	Moderate
	Residence	10.80	2	0.005	0.008	0.117	Weak
	Age group	45.71	4	<0.001	<0.001	0.181	Weak
	Education	17.51	4	0.002	0.003	0.103	Weak
	Income	71.67	8	<0.001	<0.001	0.223	Moderate
	Occupation ^a^	101.92	12	<0.001	<0.001	0.265	Moderate
	Hunting connection	82.57	4	<0.001	<0.001	0.248	Moderate
**Importance of origin (*n* = 640)**	Sex	1.70	3	0.636	0.696	0.000	Negligible
	Residence	4.12	3	0.249	0.299	0.042	Negligible
	Age group	28.84	6	<0.001	<0.001	0.134	Weak
	Education	9.62	6	0.142	0.177	0.053	Negligible
	Income	17.75	12	0.124	0.173	0.055	Negligible
	Occupation ^a^	33.73	18	0.018	0.028	0.091	Negligible
	Hunting connection	29.16	6	<0.001	<0.001	0.135	Weak

Legend: χ^2^, statistics and df are from Pearson’s chi-square tests. For rows marked ^a^, the reported *p*-value is the Fisher–Freeman–Halton exact-test *p*-value; the displayed Pearson χ^2^, statistic and df are retained for descriptive reference only and are not the basis of inference. The exact *p*-value was used for multiplicity adjustment. Benjamini–Hochberg correction was applied across the 35 comparisons.

**Table 5:** Group differences in self-rated information about game meat.

Grouping	Test	Statistic	df	*p*	Effect (Type)	Value
Sex	Mann–Whitney U	63,524.50	–	<0.001 ***	rank-biserial *r*	0.296
Residence	Mann–Whitney U	57,576.50	–	<0.001 ***	rank-biserial *r*	0.202
Age group	Kruskal–Wallis	43.23	2	<0.001 ***	ε^2^	0.065
Education	Kruskal–Wallis	6.71	2	0.035 *	ε^2^	0.007
Income	Kruskal–Wallis	33.52	4	<0.001 ***	ε^2^	0.046
Occupation	Kruskal–Wallis	119.50	6	<0.001 ***	ε^2^	0.179
Hunting connection	Kruskal–Wallis	183.95	2	<0.001 ***	ε^2^	0.286

Legend: Self-rated information was measured on a 1–5 ordinal scale (1 = not informed at all; 5 = very well informed), with higher scores indicating greater perceived information about game meat. Mann–Whitney U tests were used for two-category variables and Kruskal–Wallis tests for variables with three or more categories. df, degrees of freedom; *p*, *p*-value; r, rank-biserial correlation; ε^2^, epsilon-squared effect size. * *p* < 0.05; *** *p* < 0.001.

**Table 6:** Characteristics of the three exploratory respondent profiles identified by MCA and k-means clustering.

Characteristic	Engaged (*n* = 234)	Ambivalent (*n* = 222)	Disengaged (*n* = 184)
**Self-rated information, mean**	3.5	2.4	1.7
**Previous consumption, %**	94.9	70.3	58.2
**Knows where to buy, %**	66.2	20.3	6.5
**Hunting connection, %**	63.7	25.2	22.3
**Healthy, %**	70.9	26.1	14.7
**Unsafe, %**	0.9	26.1	22.3
**Sustainable, %**	31.2	6.3	6.5
**Distrust provenance, %**	1.7	32.9	28.8

Legend: MCA, multiple correspondence analysis; *n*, number of respondents assigned to each profile. Self-rated information, knowledge of where to buy game meat, perceived healthiness, perceived unsafety, perceived sustainability, and distrust of provenance were derived from variables used as active inputs in the MCA and therefore partly contribute to the construction of the profiles themselves. Previous consumption and hunting connection were not active MCA variables and are shown as external descriptive characteristics.

**Table 7:** Multivariable logistic regression models of factors associated with previous game-meat consumption and willingness to try game meat.

**Panel A. Previous Game-Meat Consumption (*n* = 640)** ***McFadden pseudo-R^2^ = 0.205; likelihood-ratio p < 0.001; Hosmer–Lemeshow χ^2^ (8) = 8.53, p = 0.384; maximum VIF = 1.78.***			
**Variable**	**OR**	**95% CI**	***p***
**Male sex**	1.57	0.98–2.52	0.060
**Rural residence**	0.83	0.53–1.30	0.419
**Age 25–34 years**	**2.20**	**1.28–3.78**	**0.004**
**Age ≥ 35 years**	**3.10**	**1.53–6.28**	**0.002**
**Higher education**	1.00	0.61–1.62	0.987
**Postgraduate education**	1.29	0.43–3.82	0.651
**Any personal income (vs. none)**	1.16	0.74–1.82	0.526
**Family/friends involved in hunting**	**4.81**	**2.59–8.94**	**<0.001**
**Active hunter** (hunting connection)	2.98	0.64–13.92	0.164
**Self-rated information, per 1-point increase**	**1.77**	**1.41–2.22**	**<0.001**
**Panel B. Willingness to Try Game Meat Among Non-Consumers (*n* = 152)** ***McFadden pseudo-R^2^ = 0.068; likelihood-ratio p = 0.045; Hosmer–Lemeshow χ^2^ (8) = 1.74, p = 0.988; maximum VIF = 1.20.***			
**Variable**	**OR**	**95% CI**	***p***
**Male sex**	**3.59**	**1.52–8.48**	**0.003**
**Rural residence**	1.16	0.56–2.41	0.682
**Age 25–34 years**	2.04	0.73–5.64	0.172
**Age ≥ 35 years**	0.73	0.21–2.53	0.615
**Any personal income (vs. none)**	1.10	0.53–2.26	0.801
**Any hunting connection**	0.58	0.19–1.84	0.360
**Self-rated information, per 1-point increase**	1.01	0.69–1.49	0.952

Legend: OR, odds ratio; CI, confidence interval; VIF, variance inflation factor. Reference categories were female sex, urban residence, age 18–24 years, and no hunting connection; for education in Panel A, high-school education or lower was the reference category. Self-rated information was entered as a continuous 1–5 score. Bold values indicate statistically significant associations (*p* < 0.05). The “Active hunter (hunting connection)” predictor in Panel A derives from the hunting-connection variable and is distinct from the occupational category “Active hunter (occupation)” reported in Table 1.

**Table 8:** Reported barriers to game-meat consumption according to previous consumption experience.

Barrier	Non-Consumers n	Non-Consumers, %	Previous Consumption + Barrier Response, n	Previous Consumption + Barrier Response, %	φ	Adjusted *p* ^a^
**No opportunity/availability**	96	63.2	80	37.2	0.256	<0.001 ***
**Do not like the idea of eating wild animals**	66	43.4	49	22.8	0.219	<0.001 ***
**Consider hunting unethical**	52	34.2	47	21.9	0.137	0.052
**Fear of contamination/disease**	32	21.1	53	24.7	0.042	1.000
**Consider it difficult to cook**	30	19.7	71	33.0	0.147	0.030 *
**Perceived as too expensive**	26	17.1	57	26.5	0.111	0.203

Legend: *n*, number of respondents selecting each barrier; φ, phi coefficient. The no-previous-consumption group included respondents who had never consumed game meat (*n* = 152), whereas the previous-consumption group included respondents who had consumed game meat but also completed the barrier item (*n* = 215). Multiple responses were allowed; therefore, percentages may sum to more than 100%. *p*-values were Bonferroni-adjusted for six comparisons. ^a^ Bonferroni-adjusted *p*-value for the between-group difference; * *p* < 0.05; *** *p* < 0.001.

## Data Availability

The original contributions presented in this study are included in the article/Supplementary Materials. Further inquiries can be directed to the corresponding authors.

## Supplementary Materials

The following supporting information can be downloaded at: https://www.mdpi.com/article/10.3390/foods15183179/s1. The following supporting information is available online: Data S1: Raw survey data (*n* = 640); File S1: English translation of the questionnaire; File S2: STROBE reporting checklist; File S3: CHERRIES reporting checklist; Table S1: Dunn’s post-hoc pairwise comparisons of self-rated information across sociodemographic groups following significant Kruskal–Wallis tests; Table S2: Active variables and categories included in the multiple correspondence analysis (MCA); Table S3: Sensitivity analysis of the functional form of self-rated information in the logistic regression models.
