# Supplementary material for: Consumer Attitudes, Knowledge, and Consumption Behaviours Toward Game Meat in Romania: Evidence from an Online Convenience Sample
Source: Foods. 2026 Sep 8;15(18):3179. doi: 10.3390/foods15183179 (PMC13605801; doi:10.3390/foods15183179)
Supplement: Supplementary file 1 [file foods-15-03179-s001.zip › foods-4507377-supplementary/File S1. English translation of the questionnaire.pdf]

Questionnaire — Consumer Perceptions of Game Meat (Romania)

Academic English translation. Original instrument administered in Romanian (*Percepția consumatorilor asupra cărnii de vânat*). Response options are reproduced exactly as presented to respondents; the English rendering preserves the original meaning and scale anchors. This document doubles as the Supplementary questionnaire and as the source of the English variable labels used throughout the analysis.

Item types: **SC** = single choice; **MC** = multiple choice ("select all that apply"); **MC≤3** = multiple choice capped at three selections; **OS** = ordinal scale; **FT** = free text.

Section 1 — Sociodemographic profile

1.1. Sex *(SC)* — RO: *Sex*

- Female — *Feminin*
- Male — *Masculin*

1.2. Age *(SC, ordinal)* — RO: *Vârsta*

- < 18 — *< 18*
- 18–24 — *18-24*
- 25–34 — *25-34*

- 35–44 — *35-44*

- 45–54 — *45-54*

- 55–64 — *55-64*

- 65+ — *65+*

1.3. Area of residence *(SC)* — RO: *Mediul de rezidență*

- Urban — *Urban*

- Rural — *Rural*

1.4. Education level *(SC, ordinal)* — RO: *Nivel de educație*

- No formal education — *Fără studii*

- Primary/lower-secondary — *Studii primare/gimnaziale*

- High school — *Liceu*

- Higher education (university) — *Studii superioare*

- Postgraduate studies — *Studii postuniversitare*

1.5. Personal monthly income *(SC, ordinal)* — RO: *Venit lunar personal*

- No personal income — *Nu am venit personal*

- Under 2000 RON — *Sub 2000 ron*

- 2000–4000 RON — *2000 - 4000 ron*

- 4001–6000 RON — *4001 - 6000 ron*

- Over 7000 RON — *Peste 7000 ron*

1.6. What is your current occupation? *(SC)* — RO: *Care este ocupația dumneavoastră actuală?*

- Student — *Student(ă)*

- Employed in another field — *Angajat(ă) în alt domeniu*

- Active hunter (member of a hunting association) — *Vânător activ (membru într-o asociație de vânătoare)*

- Working in the food/agri-food sector — *Lucrez în domeniul alimentar/agroalimentar*

- Entrepreneur — *Antreprenor*

- Unemployed / seeking work — *Șomer(ă) / în căutarea unui loc de muncă*

- Retired — *Pensionar*

1.7. Do you have direct links to hunting activity? *(SC)* — RO: *Aveți legături directe cu activitatea de vânătoare?*

- No connection to the field — *Nu am nicio legătură cu domeniul*

- Family members/friends who are hunters — *Am membri ai familiei/prieteni care sunt vânători*

- I am an active hunter — *Sunt vânător activ*

Section 2 — Awareness and knowledge

2.1. Have you heard of the following types of game meat? (select all that apply) *(MC)* — RO: *Ai auzit de următoarele tipuri de carne de vânat?*

- Red deer — *Cerb*

- Roe deer — *Căprior*

- Wild boar — *Mistreț*

- Pheasant — *Fazan*

- Other — *Altele*

2.2. Where did you learn about game meat? (select all that apply) *(MC)* — RO: *De unde ai aflat despre carnea de vânat?*

- Family/friends — *Familie/prieteni*

- Restaurants/retail — *Restaurante/Comerț*

- Mass media (TV, press) — *Mass-media (TV, presă)*

- Internet/social media — *Internet/rețele sociale*

- Hunters / hunting associations — *Vânători/ Asociații de vânătoare*

- I do not remember — *Nu îmi amintesc*

2.3. Do you know where game meat or hunting products can be bought in your area? *(SC)* — RO: *Știi unde se poate cumpăra carne de vânat sau produse vânătorești în zona ta?*

- Yes — *Da*

- No — *Nu*

- Not interested — *Nu sunt interesat(ă)*

2.4. On a scale from 1 to 5, how well informed do you consider yourself about game meat? *(OS, 1–5)* — RO: *Pe o scară de la 1 la 5, cât de informat(ă) te consideri în legătură cu carnea de vânat?*

- 1 (not at all) — *1 (deloc)*

- 2, 3, 4

- 5 (very well informed) — *5 (foarte bine informat)*

2.5. How important is the origin of the meat to you? *(SC, ordinal)* — RO: *Cât de importantă este pentru tine originea cărnii?*

- Very important — *Foarte importantă*

- Important — *Importantă*

- Indifferent — *Indiferentă*

- I have never thought about it — *Nu m-am gândit niciodată la asta*

Section 3 — Perceptions and attitudes

3.1. Which characteristics do you associate with game meat? (select all that apply) *(MC)* — RO: *Ce caracteristici asociezi cărnii de vânat?*

- Healthy — *Sănătoasă*

- Natural/ecological — *Naturală/Ecologică*

- Tasty/refined — *Gustoasă/Rafinată*

- Traditional — *Tradițională*

- Luxury — *De lux*

- Difficult to prepare — *Dificil de preparat*

- Dangerous (possibly contaminated) — *Periculoasă (posibil contaminată)*

- Unsafe — *Nesigură*

3.2. In terms of price, how do you consider game meat/hunting products to be? *(SC, ordinal)* — RO: *Din punct de vedere al prețului cum consideri că este carnea de vânat/produsele vânătorești?*

- Affordable for my budget — *Accesibilă pentru bugetul meu*

- Moderately priced — *Preț moderat*

- Expensive for my budget — *Scumpă pentru bugetul meu*

3.3. Select from the list only the statements you agree with (select up to 3) *(MC≤3)* — RO: *Alegeți din lista de mai jos doar afirmațiile față de care sunteți de acord (bifează maxim 3)*

- I would like to consume game meat more often — *Mi-ar plăcea să consum carne de vânat mai des*

- Game meat is hard to find in retail — *Carnea de vânat este greu de găsit în comerț*

- Preparing game meat is complicated — *Prepararea cărnii de vânat este complicată*

- Game meat is healthier than meat from domestic animals — *Carnea de vânat este mai sănătoasă decât cea a animalelor domestice*

- It is a sustainable choice for modern nutrition — *Este o alegere sustenabilă pentru alimentația modernă*

- I am concerned about the ethics of hunting — *Mă preocupă etica vânătorii*

- I do not trust the provenance of game meat — *Nu am încredere în proveniența cărnii de vânat*

Section 4 — Consumption behavior *(asked of respondents who have consumed game meat)*

4.1. Have you ever consumed game meat or other hunting products? *(SC — gate item)* — RO: *Ai consumat vreodată carne de vânat sau alte produse vânătorești?*

- Yes — *Da*

- No (if you selected this, skip directly to Section 5) — *Nu (dacă ați ales acest răspuns treceți direct la secțiunea 5)*

4.2. Which types of game meat have you consumed? (select all that apply) *(MC)* — RO: *Ce tipuri de carne de vânat ai consumat?*

- Red deer — *Cerb* · Roe deer — *Căprior* · Wild boar — *Mistreț* · Pheasant — *Fazan* · Other — *Altele*

4.3. If you selected "Other" above, specify the type of game meat (e.g., bear, wild birds, hare, etc.) *(FT)* — RO: *Dacă la întrebarea anterioară ați ales varianta "Altele" scrieți mai jos la ce tip de carne de vânat faceți referire*

4.4. Which types of hunting products have you consumed? (select several if applicable) *(MC)* — RO: *Ce tip de produse vânătorești ai consumat?*

- Fresh meat (chilled or frozen) — *Carne proaspătă (refrigerată sau congelată)*

- Processed meat (smoked, dried, marinated, etc.) — *Carne preparată (afumată, uscată, marinate etc.)*

- Semi-processed products (sausages, pâtés, canned goods, salami, etc.) — *Semipreparate (cârnați, pateuri, conserve, salamuri, etc.)*

- All of the above — *Toate de mai sus*

4.5. Where have you consumed game meat? (select several) *(MC)* — RO: *Unde ai consumat carne de vânat?*

- At home (family) — *Acasă (familie)*

- At friends'/relatives' — *La prieteni/rude*

- At a restaurant — *La restaurant*

- At an event (hunt/festival/wedding, etc.) — *La un eveniment (vânătoare/festival/nuntă etc.)*

- All of the above — *Toate de mai sus*

4.6. How often do you consume game meat? *(SC, ordinal)* — RO: *Cât de des consumi carne de vânat?*

- Weekly — *Săptămânal*

- Monthly — *Lunar*

- A few times a year — *De câteva ori pe an*

- Once a year or less — *O dată pe an sau mai rar*

- Only occasionally — *Doar ocazional*

4.7. Where did you buy/receive the game meat? (select several) *(MC)* — RO: *De unde ai cumpărat / primit carnea de vânat?*

- Directly from hunters/friends — *Direct de la vânători/prieteni*

- Specialized shops — *Magazine specializate*

- Traditional markets — *Piețe tradiționale*

- Restaurants — *Restaurante*

4.8. What would encourage you to consume game meat more often? (select up to 3) *(MC≤3)* — RO: *Ce te-ar încuraja să consumi mai des carne de vânat?*

- Lower price — *Preț mai mic*

- Availability in shops/markets — *Accesibilitate în magazine/piețe*

- Recipes and cooking guides — *Rețete și ghiduri de gătit*

- Recommendations from doctors/nutritionists — *Recomandări din partea medicilor/nutriționiștilor*

- Information campaigns — *Campanii de informare*

Section 5 — Non-consumption and future intention

5.1. If you do not consume game meat, what are the main reasons? (up to 3) *(MC≤3)* — RO: *Dacă nu consumi carne de vânat, care sunt principalele motive?*

- I have not had the opportunity — *Nu am avut ocazia*

- It is expensive — *Este scumpă*

- I do not know how to prepare it / it is hard to cook — *Nu știu cum se prepară/este greu de gătit*

- I am afraid of contamination — *Îmi este teamă de contaminare*

- I do not like the idea of eating wild animals — *Nu îmi place ideea de a mânca animale sălbatice*

- I consider hunting to be immoral — *Consider că vânătoarea este imorală*

5.2. Would you be willing to try game meat in the future? *(SC)* — RO: *Ai fi dispus(ă) să încerci carne de vânat în viitor?*

- Yes — *Da*

- No — *Nu*
