# Supplementary material for: Consumer Attitudes, Knowledge, and Consumption Behaviours Toward Game Meat in Romania: Evidence from an Online Convenience Sample
Source: Foods. 2026 Sep 8;15(18):3179. doi: 10.3390/foods15183179 (PMC13605801; doi:10.3390/foods15183179)
Supplement: Supplementary file 1 [file foods-15-03179-s001.zip › foods-4507377-supplementary/File S2. STROBE reporting checklist.pdf]

File S2. STROBE Reporting Checklist

Section	Item	Reporting criterion (brief)	Location in manuscript	Status / manuscript note
**Title and abstract**	1a	Identify the study design in the title or abstract.	Title; Abstract; Section 2.1	Reported. The title identifies an online convenience sample; the Abstract and Section 2.1 identify the cross-sectional online survey design.
**Title and abstract**	1b	Provide a balanced abstract summarising methods and main findings.	Abstract	Reported. The Abstract summarises the sample, exclusions, analytical methods, principal findings, limitations, and cautious interpretation.
**Background/rationale**	2	Explain the scientific background and rationale.	Section 1, Introduction	Reported.
**Objectives**	3	State specific objectives and any prespecified hypotheses.	Section 1, final two paragraphs	Reported. Five study objectives are stated. No formal causal hypotheses were prespecified.
**Study design**	4	Present key design features early in the manuscript.	Section 2.1	Reported. Cross-sectional, self-administered online non-probability convenience survey.
**Setting**	5	Describe setting, locations, and relevant recruitment/data-collection dates.	Section 2.1	Reported. The study was conducted in Romania. Recruitment used university-associated staff/student networks in Timișoara, București, Cluj-Napoca, and Iași plus digital social/messaging networks. The field period was 13 May 2025 to 3 May 2026.
**Participants**	6a	Describe eligibility criteria and participant selection/recruitment.	Section 2.1	Reported. Eligible participants were Romanian residents aged >=18 years. Recruitment used a non-probability convenience approach. Of 643 complete records, 3 respondents aged <18 years were excluded, leaving N=640.
**Variables**	7	Define outcomes, predictors/exposures, and other variables as relevant.	Sections 2.2-2.4; File S1	Reported. Questionnaire domains, variable measurement levels, binary outcomes, regression predictors, and MCA inputs are defined.
**Data sources / measurement**	8	Describe data sources and assessment methods for variables.	Section 2.2; File S1	Reported. The Romanian questionnaire, literature-informed development, expert review, pilot test (n=20), response formats, conditional routing, translation/reconciliation, and full English questionnaire are described.
**Bias**	9	Describe efforts to address or acknowledge potential sources of bias.	Sections 2.1 and 4.6	Reported. The manuscript addresses convenience-sampling and recruitment-channel bias, self-report/recall bias, cross-sectional directionality, hunting-network selection, and questionnaire-design limitations.
**Study size**	10	Explain how the study size was determined.	Section 2.1; Sections 3.7 and 4.6	Reported. No formal a priori power calculation was performed; sample size followed recruitment feasibility. The smaller willingness subgroup (n=152; 76 willing) is interpreted cautiously.
**Quantitative variables**	11	Explain handling/grouping of quantitative or ordinal variables and reasons for categorisation.	Sections 2.3-2.4	Reported. Age and education categories were collapsed for sparse cells; income was dichotomised for multivariable models; self-rated information was treated ordinally for group comparisons and as a one-point predictor with sensitivity analysis in logistic models.
**Statistical methods**	12a	Describe statistical methods, including multivariable adjustment.	Section 2.4	Reported. Chi-square/exact tests, FDR and Bonferroni corrections, non-parametric tests, Dunn post-hoc tests, MCA/clustering, logistic regression, VIF, model-fit and calibration diagnostics are described.
**Statistical methods**	12b	Describe subgroup and interaction analyses.	Sections 2.2 and 2.4	Reported. Conditional analyses among previous consumers/non-consumers and the exploratory barrier comparison are described. No interaction models were prespecified.
**Statistical methods**	12c	Explain handling of missing or non-applicable data.	Section 2.2; Tables 2-3	Reported. Conditional questions use applicable valid-response denominators; logically inapplicable responses were excluded; item-specific n values are reported; the highly incomplete free-text item was excluded from quantitative analysis.
**Statistical methods**	12d	If relevant, describe methods accounting for the sampling strategy.	Sections 2.1 and 2.4	No survey weighting was applied to the non-probability convenience sample. Results are reported for the analytical sample and explicitly not as nationally representative estimates.
**Statistical methods**	12e	Describe sensitivity analyses.	Sections 2.4, 3.6 and 3.7	Reported. MCA/clustering stability was assessed using additional dimensions and resampling. Logistic models were refitted with self-rated

				information as a categorical predictor and compared with linear-score specifications by likelihood-ratio tests.
**Participants**	13a	Report participant numbers at each study stage.	Sections 2.1 and 3.1	Reported. 643 complete records were obtained, 3 age-ineligible records were excluded, and 640 respondents were analysed.
**Participants**	13b	Give reasons for non-participation/exclusion when available.	Sections 2.1 and 3.1	Reported for exclusions. The number invited/viewing/starting the distributed survey was not available because recruitment did not use a defined sampling frame.
**Participants**	13c	Consider a participant-flow diagram.	Sections 2.1 and 3.1	A separate diagram was not necessary for the simple flow; the sequence 643 complete records -> 3 excluded -> 640 analysed is reported narratively.
**Descriptive data**	14a	Report participant characteristics and relevant exposures/confounders.	Section 3.1; Table 1	Reported.
**Descriptive data**	14b	Report missing-data counts for variables of interest.	Sections 3.2-3.3; Tables 2-3	Reported through item-specific denominators for conditional and valid-response subsets.
**Outcome data**	15	Report outcome events or summary measures.	Sections 3.2-3.8; Tables 2-8	Reported.
**Main results**	16a	Report unadjusted and adjusted estimates with precision; specify adjustment variables.	Sections 3.4 and 3.7; Tables 4 and 7	Reported. Bivariate associations/effect sizes and adjusted odds ratios with 95% confidence intervals are provided; model covariates and reference categories are specified.
**Main results**	16b	Report category boundaries when continuous/ordinal variables are categorised.	Sections 2.4 and 3.1; Tables 1, 4, 7	Reported.
**Main results**	16c	Where relevant, translate relative measures into absolute risk over a meaningful period.	Not applicable	Not applicable to this cross-sectional survey. Absolute sample proportions are reported directly.
**Other analyses**	17	Report subgroup, interaction, and sensitivity analyses.	Sections 3.4-3.8; Table S1; Table S2	Reported. Subgroup analyses, Dunn pairwise comparisons (Table S1), MCA inputs/categories (Table S2), MCA stability analyses, and logistic sensitivity analyses are provided.
**Key results**	18	Summarise key results with reference to objectives.	Section 4, opening and subsections	Reported.
**Limitations**	19	Discuss limitations, potential bias, and imprecision.	Section 4.6	Reported. Limitations include non-representativeness, veterinary/academic recruitment-channel effects, cross-sectional directionality, self-report, psychometric limitations, barrier-item interpretation, the income-band gap, and incomplete free-text responses; the willingness model is interpreted cautiously because of its smaller subgroup.
**Interpretation**	20	Provide cautious overall interpretation considering objectives, limitations, multiplicity, and related evidence.	Sections 4.1-4.5 and 5	Reported. Causal and population-level claims are avoided; implications are framed as hypotheses/priorities for future evaluation.
**Generalisability**	21	Discuss external validity/generalisation.	Sections 2.1, 4.6 and 5	Reported. Findings are explicitly limited to the surveyed young/student-dominated convenience sample and are not presented as national estimates.
**Funding**	22	Report funding source and role of funders.	Funding Statement	Reported. The study received no external funding.

Reference: von Elm, E.; Altman, D.G.; Egger, M.; Pocock, S.J.; Gøtzsche, P.C.; Vandenbroucke, J.P.; STROBE Initiative. The Strengthening the Reporting of Observational Studies in Epidemiology (STROBE) statement: guidelines for reporting observational studies. PLoS Med. 2007, 4, e296. https://doi.org/10.1371/journal.pmed.0040296
