# Supplementary material for: Consumer Attitudes, Knowledge, and Consumption Behaviours Toward Game Meat in Romania: Evidence from an Online Convenience Sample
Source: Foods. 2026 Sep 8;15(18):3179. doi: 10.3390/foods15183179 (PMC13605801; doi:10.3390/foods15183179)
Supplement: Supplementary file 1 [file foods-15-03179-s001.zip › foods-4507377-supplementary/File S3. CHERRIES reporting checklist.pdf]

File S3. CHERRIES Reporting Checklist

CHERRIES domain	Checklist item	Study-specific reporting	Location in manuscript / supplement
**Design**	Describe survey design.	Cross-sectional, self-administered online convenience survey conducted in Romania using Google Forms.	Abstract; Section 2.1
**IRB approval and informed consent**	IRB approval.	Formal ethical approval was not required, as confirmed by the Bioethics Commission of the University of Life Sciences "King Mihai I" from Timișoara (Decision No. 754/17.07.2026).	Institutional Review Board Statement
**IRB approval and informed consent**	Informed consent.	Participation was voluntary and anonymous. All respondents provided informed consent before beginning the questionnaire.	Section 2.1; Informed Consent Statement
**IRB approval and informed consent**	Data protection.	The survey was anonymous and did not collect sensitive personal or medical information. The analytical dataset was de-identified.	Section 2.1; Institutional Review Board Statement; Data Availability Statement
**Development and pre-testing**	Development and testing.	Questionnaire development was informed by recent European literature. The draft was reviewed for content and face validity by colleagues with relevant expertise and pilot-tested in a convenience sample of 20 consumers and non-consumers. Minor linguistic and layout changes were made before launch.	Section 2.2
**Recruitment process and description of sample**	Open survey versus closed survey.	The survey link was distributed through university-associated staff and student networks and further circulated through digital social and messaging networks. It was not administered to a fixed sampling frame or closed research panel.	Section 2.1
**Recruitment process and description of sample**	Contact mode.	Participants were reached through electronically distributed survey links.	Section 2.1
**Recruitment process and description of sample**	Advertising the survey.	The Google Forms link was circulated through university-associated networks, including veterinary education centres in Timișoara, București, Cluj-Napoca, and Iași, and through digital social and messaging networks.	Section 2.1
**Survey administration**	Web/E-mail.	The questionnaire was administered on the web using Google Forms; recruitment messages contained a link to the survey.	Section 2.1
**Survey administration**	Context.	The questionnaire concerned attitudes, knowledge, perceptions, consumption behaviour, acquisition, barriers, and willingness related to game meat.	Sections 2.1–2.2
**Survey administration**	Mandatory/voluntary participation.	Participation in the survey was voluntary. Whether every individual questionnaire item was technically mandatory in Google Forms was not documented; item-specific valid denominators are reported where applicable.	Section 2.1; Tables 2–3
**Survey administration**	Incentives.	No incentive is reported in the study documentation or manuscript.	Not reported / not applicable
**Survey administration**	Time/date.	Data were collected between 13 May 2025 and 3 May 2026.	Section 2.1
**Survey administration**	Randomization of items or questionnaires.	No item-order randomization is reported; the structured questionnaire followed the predefined survey sequence.	Section 2.2 / File S1
**Survey administration**	Adaptive questioning.	Conditional routing was used for questions intended for specific respondent subgroups according to previous consumption experience. The questionnaire flow and conditional routing were pilot-tested before launch.	Section 2.2
**Survey administration**	Number of items.	The instrument covered nine core content areas. The complete English questionnaire is provided as File S1. A single total item count is not stated in the manuscript because several questions were conditional and/or multiple-response.	Section 2.2; File S1
**Survey administration**	Number of screens/pages.	The exact number of Google Forms screens/pages was not documented in the study records available for reporting.	Not available
**Survey administration**	Completeness check.	Google Forms yielded 643 submitted questionnaire records. Item-specific missingness and conditional applicability were handled using valid-response denominators. Three records were excluded during	Sections 2.1–2.2; Section 3.1

		eligibility screening because respondents selected the <18 years category.	
**Survey administration**	Review step.	A separate post-completion review/confirmation screen was not documented in the study records available for reporting.	Not available
**Response rates**	Unique site visitor.	The number of unique individuals who received or viewed the survey invitation was not available because the link was disseminated through digital networks rather than a defined sampling frame.	Section 2.1
**Response rates**	View rate.	Not calculable because the number of unique survey-page visitors/views was not available.	Section 2.1
**Response rates**	Participation rate.	Not calculable because the number of unique visitors or eligible invitees was not available.	Section 2.1
**Response rates**	Completion rate.	A conventional completion rate could not be calculated because the denominator of survey starts/unique visitors was not available. A total of 643 submitted questionnaire records were obtained; 640 eligible records were analysed.	Section 2.1; Section 3.1
**Preventing multiple entries**	Cookies used.	No cookie-based duplicate-control procedure is reported.	Not used / not documented
**Preventing multiple entries**	IP check.	No IP-address-based duplicate-control procedure is reported.	Not used / not documented
**Preventing multiple entries**	Log-file analysis.	No separate log-file duplicate screening is reported.	Not used / not documented
**Preventing multiple entries**	Registration / one-response restriction.	Google Forms was configured to permit only one submission per account as a measure to reduce duplicate participation.	Section 2.1
**Analysis**	Handling of incomplete questionnaires.	Analyses used the applicable valid-response subsample for conditional items, with denominators reported explicitly. Logically inapplicable responses to consumption-dependent items from respondents reporting no previous consumption were excluded.	Section 2.2; Tables 2–3
**Analysis**	Questionnaires submitted with atypical timestamps.	No exclusion based on unusually short or long completion time was reported; completion-duration data were not used as an eligibility criterion.	Not applied / not documented
**Analysis**	Statistical correction / weighting.	No population weighting was applied because the study used a non-probability convenience sample. Multiplicity correction was applied to specified inferential analyses as described in the manuscript.	Sections 2.4 and 4.6
**Analysis**	Data cleaning / recoding transparency.	Semantically identical response categories were harmonised. 'All of the above' selections for product forms and consumption settings were redistributed to their constituent categories as described in the Methods.	Section 2.2

Reference: Eysenbach, G. Improving the Quality of Web Surveys: The Checklist for Reporting Results of Internet E-Surveys (CHERRIES). J. Med. Internet Res. 2004, 6, e34.
