# Supplementary material for: Consumer Attitudes, Knowledge, and Consumption Behaviours Toward Game Meat in Romania: Evidence from an Online Convenience Sample
Source: Foods. 2026 Sep 8;15(18):3179. doi: 10.3390/foods15183179 (PMC13605801; doi:10.3390/foods15183179)
Supplement: Supplementary file 1 [file foods-15-03179-s001.zip › Table_S1.pdf]

Table S1. Dunn's post-hoc pairwise comparisons of self-rated information across sociodemographic groups following significant Kruskal–Wallis tests.

Grouping variable	Pairwise comparison	Dunn z	Unadjusted p	Bonferroni-adjusted p
**Age group**	18-24 vs 25-34	-2.562	0.0104	**0.0312**
	18-24 vs ≥35	-6.547	<0.0001	**<0.0001**
	25-34 vs ≥35	-3.518	0.0004	**0.0013**
**Education**	High school or lower vs Higher education	1.669	0.0951	0.2852
	High school or lower vs Postgraduate	-1.172	0.2412	0.7235
	Higher education vs Postgraduate	-2.250	0.0245	0.0734
**Monthly income**	No personal income vs <2000 RON	0.199	0.8426	1.0000
	No personal income vs 2000–4000 RON	-2.884	0.0039	**0.0392**
	No personal income vs 4001–6000 RON	-4.160	<0.0001	**0.0003**
	No personal income vs >7000 RON	-4.044	<0.0001	**0.0005**
	<2000 RON vs 2000–4000 RON	-2.659	0.0078	0.0784
	<2000 RON vs 4001–6000 RON	-3.752	0.0002	**0.0018**
	<2000 RON vs >7000 RON	-3.746	0.0002	**0.0018**
	2000–4000 RON vs 4001–6000 RON	-1.113	0.2658	1.0000
	2000–4000 RON vs >7000 RON	-1.363	0.1729	1.0000
	4001–6000 RON vs >7000 RON	-0.363	0.7165	1.0000
**Occupation**	Student vs Employed (other field)	-0.879	0.3793	1.0000
	Student vs Active hunter (occupation)	-10.498	<0.0001	**<0.0001**
	Student vs Food/agri-food sector	-2.292	0.0219	0.4600
	Student vs Entrepreneur	-2.322	0.0202	0.4244
	Student vs Unemployed	0.802	0.4223	1.0000
	Student vs Retired	0.129	0.8971	1.0000
	Employed (other field) vs Active hunter (occupation)	-9.306	<0.0001	**<0.0001**
	Employed (other field) vs Food/agri-food sector	-1.911	0.0560	1.0000
	Employed (other field) vs Entrepreneur	-1.941	0.0523	1.0000
	Employed (other field) vs Unemployed	1.052	0.2927	1.0000
	Employed (other field) vs Retired	0.379	0.7045	1.0000
	Active hunter (occupation) vs Food/agri-food sector	3.611	0.0003	**0.0064**
	Active hunter (occupation) vs Entrepreneur	3.584	0.0003	**0.0071**
	Active hunter (occupation) vs Unemployed	5.488	<0.0001	**<0.0001**
	Active hunter (occupation) vs Retired	4.687	<0.0001	**<0.0001**
	Food/agri-food sector vs Entrepreneur	-0.022	0.9825	1.0000

	Food/agri-food sector vs Unemployed	2.104	0.0354	0.7431
	Food/agri-food sector vs Retired	1.531	0.1258	1.0000
	Entrepreneur vs Unemployed	2.123	0.0337	0.7081
	Entrepreneur vs Retired	1.550	0.1212	1.0000
	Unemployed vs Retired	-0.467	0.6404	1.0000
**Hunting connection**	No hunting connection vs Family/friends hunters	-7.861	<0.0001	**<0.0001**
	No hunting connection vs Active hunter (hunting connection)	-12.474	<0.0001	**<0.0001**
	Family/friends hunters vs Active hunter (hunting connection)	-6.562	<0.0001	**<0.0001**

Note: Dunn's tests were performed only after a significant Kruskal–Wallis omnibus test. Bonferroni adjustment was applied separately within each grouping variable. The sign of z reflects Group 1 minus Group 2 mean ranks; a negative value indicates a lower mean rank for the first-listed group. Bold adjusted p-values indicate $p < 0.05$. Self-rated information was measured on a 1–5 ordinal scale (1 = not informed at all; 5 = very well informed).
