# Supplementary material for: Consumer Attitudes, Knowledge, and Consumption Behaviours Toward Game Meat in Romania: Evidence from an Online Convenience Sample
Source: Foods. 2026 Sep 8;15(18):3179. doi: 10.3390/foods15183179 (PMC13605801; doi:10.3390/foods15183179)
Supplement: Supplementary file 1 [file foods-15-03179-s001.zip › Table_S2.pdf]

Table S2. Active variables and categories included in the multiple correspondence analysis (MCA).

Questionnaire item	Active variable/domain	Coding used in MCA	Categories/indicators entered
**Q2.1**	Game-species awareness	Multiple response; each option entered as a binary indicator (selected/not selected)	Wild boar; Roe deer; Pheasant; Red deer; Other
**Q2.2**	Information sources	Multiple response; each option entered as a binary indicator (selected/not selected)	Family/friends; Restaurants/retail; Internet/social media; Mass media; Hunters/hunting associations; Does not remember
**Q2.3**	Knowledge of where to obtain game meat	Single-response categorical variable	No; Yes; Not interested
**Q2.4**	Self-rated information about game meat	Single-response categorical variable	1 (not informed at all); 2; 3; 4; 5 (very well informed)
**Q2.5**	Importance of meat origin	Single-response categorical variable	Indifferent; Never thought about it; Important; Very important
**Q3.1**	Perceived characteristics of game meat	Multiple response; each option entered as a binary indicator (selected/not selected)	Tasty/refined; Natural/ecological; Healthy; Difficult to prepare; Potentially contaminated/dangerous; Luxury; Traditional; Unsafe
**Q3.2**	Price perception	Single-response categorical variable	Affordable; Moderate; Expensive
**Q3.3**	Agreement statements	Multiple response; each option entered as a binary indicator (selected/not selected)	Difficult to find in retail; Preparation is complicated; Would like to consume more often; Healthier than domestic meat; Concerned about hunting ethics; Distrusts provenance; Sustainable choice

Note: Sociodemographic variables and consumption-dependent variables were not used as active MCA variables. For multiple-response domains, each listed response option was entered as a separate binary indicator with selected and not-selected levels. Single-response variables were entered using their original response categories. The MCA was conducted in the full analytical sample (N = 640).
