# Supplementary material for: Consumer Attitudes, Knowledge, and Consumption Behaviours Toward Game Meat in Romania: Evidence from an Online Convenience Sample
Source: Foods. 2026 Sep 8;15(18):3179. doi: 10.3390/foods15183179 (PMC13605801; doi:10.3390/foods15183179)
Supplement: Supplementary file 1 [file foods-15-03179-s001.zip › Table_S3.pdf]

Table S3. Sensitivity analysis of the functional form of self-rated information in the logistic regression models.

The primary models treated the five-point self-rated information item as a one-point linear predictor. For sensitivity analysis, each model was refitted with self-rated information entered as a categorical five-level factor. The categorical and linear-score specifications were compared using likelihood-ratio tests.

Outcome/model	Analytical sample	Linear model log-likelihood	Categorical model log-likelihood	LR χ^2	df	p-value	Interpretation
Previous game-meat consumption	N = 640	−281.694	−281.038	1.31	3	0.727	Categorical specification did not significantly improve model fit.
Willingness to try game meat among non-consumers	n = 152	−98.161	−96.059	4.20	3	0.240	Categorical specification did not significantly improve model fit.

Note: LR χ^2, likelihood-ratio chi-square statistic; df, degrees of freedom. The categorical specification introduced three additional parameters relative to the linear-score model. Because neither likelihood-ratio comparison was statistically significant, the more parsimonious linear-score specification was retained in the primary analyses.
